# Assessment of the health needs of Syrian refugees in Lebanon and Syria’s neighboring countries

**DOI:** 10.1186/s13031-019-0211-3

**Published:** 2019-06-27

**Authors:** Nour El Arnaout, Spencer Rutherford, Thurayya Zreik, Dana Nabulsi, Nasser Yassin, Shadi Saleh

**Affiliations:** 1 Global Health Institute, American University of Beirut, Riad El Solh, Beirut, 1107 2020 Lebanon; 2 Department of Health Management and Policy, Faculty of Health Sciences, American University of Beirut, Riad El Solh, Beirut, 1107 2020 Lebanon; 3 Issam Fares Institute for Public Policy and International Affairs, American University of Beirut, Riad El Solh, Beirut, 1107 2020 Lebanon

**Keywords:** Health, Refugees, Conflict, Migration, Syria, Lebanon, Jordan, Iraq, Turkey

## Abstract

**Background:**

Health needs of displaced Syrians in refugee hosting countries have become increasingly complex in light of the protracted Syrian conflict. The primary aim of this study was to identify the primary health needs of displaced Syrians in Iraq, Jordan, Lebanon, Turkey, and Syria.

**Methods:**

A systematic review was performed using 6 electronic databases, and multiple grey literature sources. Title, abstract, and full text screening were conducted following the Preferred Reporting Items for Systematic Reviews and Meta-Analyses. The target population was Syrian individuals displaced due to conflict in Syria and its neighboring countries. The outcomes of interest were health needs (i.e. health problems that can be addressed by health services), gaps in health services, training, and workforce. Studies on mixed refugee populations and Syrians displaced prior to the conflict were excluded.

**Results:**

The Lebanon-specific results of the review were validated through two stakeholder roundtable discussions conducted with representatives from primary healthcare centers, non-governmental organizations and humanitarian aid agencies. A total of 63 articles were included in the analysis. Mental health and women’s health were identified as the greatest health needs in the region. The most common health problems were Non-communicable diseases in Jordan, women’s health in Lebanon and mental health in Turkey. Studies addressing gaps in services found the highest gap in general healthcare services, followed by women’s health, mental health, and vaccinations. Sub-optimal training and availability of health workers was also noted particularly in Syria.

Results from the stakeholders’ discussions in Lebanon showed communicable diseases, women’s health and mental health as the main health needs of Syrian refugees in Lebanon. Reported barriers to accessing health services included geographical barriers and lack of necessary awareness and education.

**Conclusion:**

There is a need for an enhanced synchronized approach in Syria’s refugee hosting neighboring countries to reduce the existing gaps in responding to the needs of Syrian refugees, especially in regards to women’s health, mental health, and communicable diseases. This mainly includes training of healthcare workers to ensure a skilled workforce and community-based efforts to overcome barriers to access, including lack of knowledge and awareness about highly prevalent health conditions.

**Electronic supplementary material:**

The online version of this article (10.1186/s13031-019-0211-3) contains supplementary material, which is available to authorized users.

## Background

With 8 years since the onset of the severe humanitarian crisis in Syria, the effects of the conflict continue to reverberate throughout the international community. Arguably, the most significant among the conflict’s implications is the displacement of over 13 million Syrians, with an estimated 6.6 million displaced internally, and a further 5.5 million fleeing to other countries [1]. Refugee-hosting countries neighboring Syria, namely Lebanon, Jordan, Iraq, and Turkey, have been enduring the full burden of this refugee crisis. These countries currently host a combined 5 million Syrians, representing nearly 95% of the total number of registered Syrian refugees worldwide [1].

Due to the protracted nature of the conflict, the health needs of displaced Syrians have become increasingly complex to diagnose and manage. Studies have indicated that non-communicable diseases (NCDs) [2, 3], mental health disorders including post-traumatic stress disorder (PTSD) and depression [4, 5], as well as communicable diseases (CDs) such as Cutaneous Leishmaniasis, have been noted to be the most prevalent cases observed in refugee settings [6, 7]. Despite efforts invested by local and international organizations to address these cases, a comprehensive mapping of the health needs of Syrian refugees residing in Lebanon and other countries neighboring Syria is lacking. Such an assessment is crucial in order to strategize and prioritize areas of interventions to address the health needs of Syrian refugees, especially in light of the lack of funding and resources [8].

### Context of Lebanon

Lebanon in specific has the highest per capita concentration of refugees worldwide [9]. The United Nations High Commissioner for Human Rights (UNHCR) June 2018 data shows that around 1 million Syrian refugees are currently residing in Lebanon, equivalent to 25% of the Lebanese population [10]. This is in addition to around 300,000 to 500,000 unregistered Syrian refugees present in Lebanon [11]. Most of the refugee population settled in underserved rural and peri-urban areas [2, 12]. While the Lebanese healthcare system suffered from significant fragmentation prior to the influx of Syrian refugees, the dense distribution of Syrian refugees in low-resource areas, known to have sub-optimal local capacity, exposed the system to further challenges.

The response to the health needs of Syrian refugees during the past 8 years was achieved by the joint efforts of a triad of actors. These actors consist of a network of Primary Healthcare Centers (PHCs) of the Lebanese Ministry of Public Health (MOPH), local non-governmental organizations (NGOs), as well as international NGOs and humanitarian aid agencies. However, optimizing the effectiveness and efficiency of this response continues to be a challenge, due to major duplication of effort [12]. This duplication may be the result of the dearth of evidence available to the different actors, variation in health-seeking behaviors and trust in institutions among targeted populations, or the channeling of funds to only specific health issues. Worth noting is the lack of a comprehensive reliable reference triangulating available data from different sources on the priority health needs of Syrian refugees within hosting communities, including Lebanon. This in turn hinders investments in targeted interventions towards priority health areas to reduce the existing gap.

The main aim of this study is to comprehensively identify the primary health needs of displaced Syrians in refugee hosting countries including Iraq, Jordan, Lebanon, Turkey, and Syria through a systematic review of the literature. With Lebanon hosting the highest number of Syrian refugees per capita globally, the study also aims to validate the Lebanon-specific results of the systematic review and to prioritize the health areas in need of intervention through stakeholders’ engagement meetings.

## Methods

### Systematic Review of literature

#### Protocol and Registration

The protocol for the systematic review component of this study was registered in the PROSPERO prospective register of systematic reviews under registration number CRD42017079530 (https://www.crd.york.ac.uk/prospero/display_record.php?RecordID=79530.)

#### Eligibility Criteria

The systematic review included the following types of study designs: randomized, non-randomized, case-control, cohort, case-studies, cross-sectional, and qualitative. Studies using secondary data, commentaries, and opinion pieces were excluded. Only studies in English, published between 2011 and August 2017, were included.

The inclusion of target population was limited to Syrians displaced due to the conflict, in Iraq, Jordan, Lebanon, Syria and Turkey, excluding any other refugee population, and Syrians displaced prior to the conflict.

Outcomes of interest included the following:

- Health outcomes, including prevalence and incidence of specific diseases and disorders.
- Gaps in health services, such as their availability, accessibility, and comprehensiveness.
- Gaps in health training, from first aid training to professional degrees.
- Gaps in the health workforce, such as doctors, paramedics, and nurses.

#### Search Strategy

A systematic search strategy [see Additional file 1] was run on the following electronic databases: Medline, PubMed, EMBASE, Scopus, Cumulative Index to Nursing and Allied Health Literature (CINAHL), and the World Health Organization (WHO) Global Health Library [see Additional file 2]**.** The search range was from the date of the database inception until August 2017. The grey literature was also searched using databases from RefWorld, the WHO Eastern Mediterranean Regional Office (EMRO), Médecins Sans Frontières (MSF), and the International Committee of the Red Cross (ICRC). The terms “health”, “practitioner”, and “refugee” among others were used for the grey literature search which was restricted to Iraq, Jordan, Lebanon, Syria, and Turkey [see Additional file 3]. The search was not restricted to a specific language or date range.

#### Definition of Search Terms

Three overarching concepts were used in the search strategy of the systematic review of literature: health (the outcome), refugees (the population), and the refugee-hosting countries neighboring Syria (the region). Within the first concept of “health”, the aim was to capture both the demand side (health needs) and the gaps in the supply side (health service delivery, health workforce, and health training). “Health needs” are defined in our review by a narrower definition that only includes health problems which can be addressed by health services [13]. Consequently, factors that are important to health, but not addressable within the boundaries of health services, such as shelter, education, and employment, were not included. On the supply side, health service delivery is represented by access, availability, utilization, and coverage; all of which indicate whether or not the target population are receiving the services they need [14]. In parallel, the health workforce is defined as those engaged in actions that primarily intend to enhance health including both clinical staff (e.g. physicians, nurses, pharmacists, and dentists) as well as management and support staff [14]. As for health training, it relates to the capacity building and education of the health workforce involved in service delivery [14].

As Lebanon, Jordan, and Iraq are non-signatories to the 1951 Convention Relating to the Status of Refugees [15], the definition for “refugee” had to be broadened to include any Syrian individual displaced by the Syrian conflict in Iraq, Jordan, Lebanon, Syria, or Turkey, including terms such as “asylum seeker”, “displaced individual”, and “migrant”, to name a few.

#### Screening and Selection Process

Before the selection process, duplicates and studies published prior to 2011 were removed. The selection process was conducted in two stages; the title and abstract screening stage, and the full-text screening stage. In the first stage, two reviewers independently screened the title and abstract of identified citations for eligibility. In the second stage, full texts of citations that were judged as potentially eligible by at least one reviewer were screened by the two reviewers independently using the same eligibility criteria. Results were then compared and disagreements were resolved by discussion or by consulting a third reviewer. Agreement level between reviewers was calculated using the kappa statistic.

The grey literature databases were screened by one reviewer following the same set of eligibility criteria. Texts that fulfilled the eligibility criteria were then screened by two reviewers independently. Results were compared, and disagreements resolved.

#### Data Abstraction Process

Data was collected in a data abstraction table including the following information: study design; publication date of the study; country and setting where the study was conducted; sample size, age and sex distribution; population subtype (i.e. women, children, elderly), if any, and; which research questions the study addressed (health needs, gaps in health services, gap in capacity building and training, or gap in availability of health workforce). Findings relevant to each of the research questions were abstracted from each included study.

#### Risk of Bias Assessment

Risk of bias was assessed using the McGill Mixed Methods Appraisal Tool (MMAT) at the study level [16]. One reviewer worked independently to assess the quality of the study based on 1) the measurements, 2) what was reported, 3) the representativeness of the data, and 4) the risk of bias of the study which was based on potential selection bias, considerations regarding contextual factors, and the researchers influence.

#### Data Synthesis

Results from the data abstraction were reported narratively organized by the research question and further subdivided by country. Results were further analyzed differentially depending on the research question and stratified based on themes. Gaps in health service delivery and workforce availability and training were presented in a tabular format for each country separately. The grey literature findings were reported narratively.

### Validation of Lebanon-Specific Results

To ensure a contextualized assessment of the health needs of Syrian refugees in Lebanon, a validation of the results of the Lebanon-specific results of the systematic review was conducted through two stakeholders’ roundtable discussions.

The first validation meeting gathered six designated stakeholders representing PHCs belonging to the National PHC Network of the MOPH. The research team coordinated with the Head of the Social Health & the Primary Health Care department at the MOPH in Lebanon to arrange the stakeholders’ roundtable discussion at the ministry, gathering PHCs directors/representatives of selected PHCs, recognized by having the highest load of Syrian refugee patients, and ensuring the presence of at least one representative from each of Bekaa, North Lebanon, Beirut, and South areas of Lebanon. Lebanon-specific results of the systematic review arranged in a format of visually appealing figures were presented to the attendees. PHC representatives were subsequently asked to allocate to each of the health needs presented, a priority index indicating the extent to which they perceive that the stated health area has a priority over others to be addressed among refugees and host communities. The priority index takes into account both the needs of refugees to enhanced services in a stated health need and the existing lack in the availability of services and skilled health personnel addressing these health needs.

For the second validation meeting, invitations to participate in the validation meeting were sent via email to 43 representatives of leading local and international NGOs and humanitarian aid agencies directly involved in the provision of health services to refugees residing in different regions across Lebanon. Twenty five representatives confirmed attendance and participated in the discussion. The meeting, which was attended by heads of missions of NGOs and humanitarian agencies, as well as heads of delegations, country coordinators, and health coordinators, among others, was facilitated by the project coordinator. Attendees were first asked to share and exchange information about their current activities in the delivery of health services to Syrian refugees in Lebanon as well as gaps in services being provided and areas in need of intervention. Next, the Lebanon-specific results of the systematic review were presented in a single-blinded manner (i.e. the NGOs representatives were not presented with the data on how the PHCs representatives of the first validation meeting ranked the health needs by priority and vice versa) in order to decrease potential sources of biases. Representatives were randomly divided into 4 working groups and asked to rank the presented health needs using a priority index to indicate which of these needs they perceive as high priority areas in need of intervention.

Results from the two roundtable discussions were documented and extensive notes were taken during the meeting by a note taker.

## Results

### Systematic Review of Literature

#### Study Selection

**Fig. 1:**
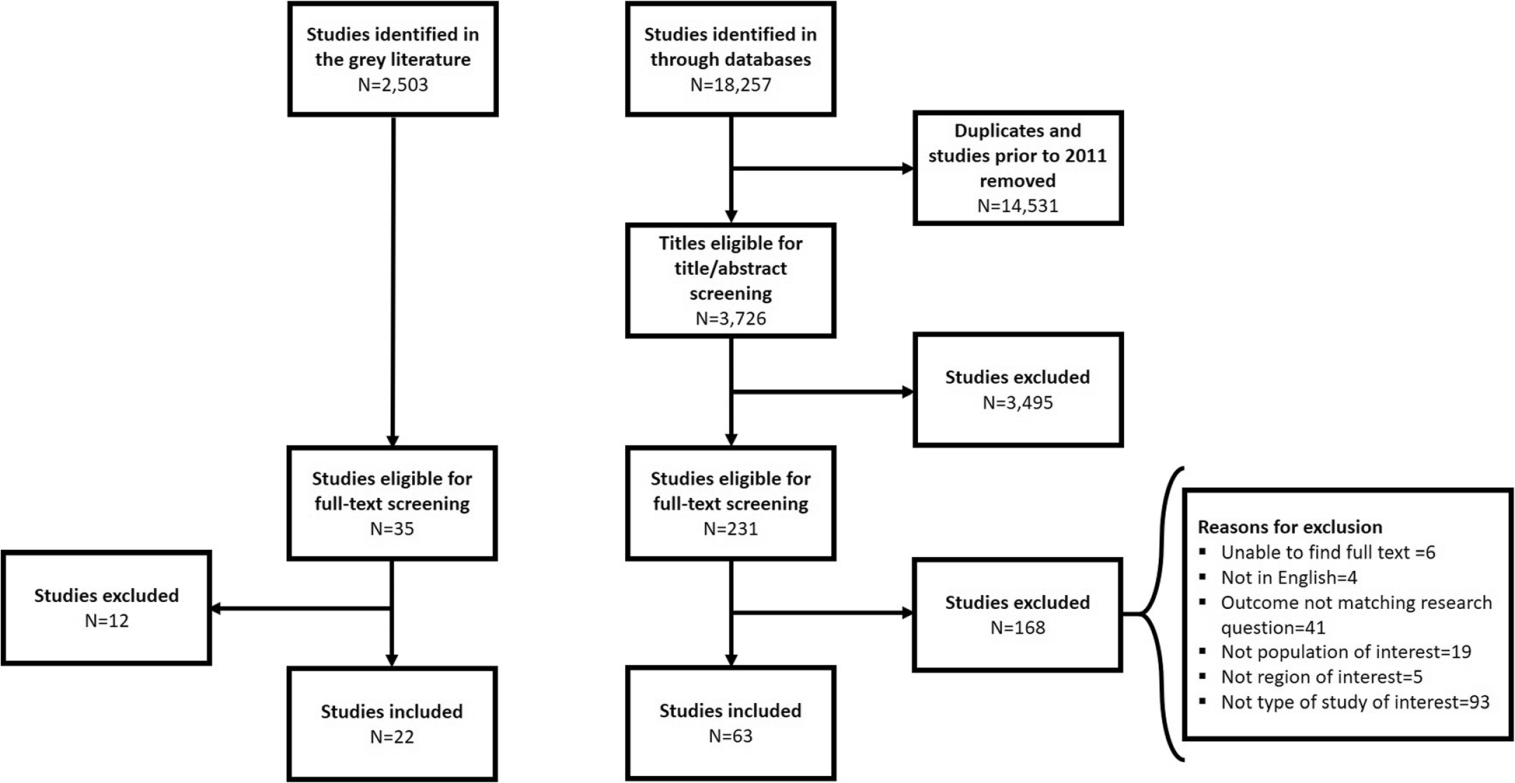
Preferred Reporting Items for Systematic Reviews and Meta-Analyses (PRISMA) flow diagram

The selection process is depicted in Fig. 1 using the Preferred Reporting Items for Systematic Reviews and Meta-Analyses (PRISMA) flow diagram. Out of 18,257 articles obtained as a result of the systematic search on electronic databases, 3726 studies were eligible for title and abstract screening following removal of duplicates and pre-conflict studies. Two hundred thirty-one studies were selected for full-text screening, and 63 articles were included in the analysis of this review. Rejected studies are outlined in Additional file 4. Agreement between reviewers was calculated using the kappa statistic which was found to be 0.92, indicating a very high level of agreement [17].

As for the grey literature searches, 35 out of 2503 resources were related to the research questions, of which 22 references were concurrent with the eligibility criteria.

#### Characteristics of Included Studies

Additional file 5 provides details of the 63 included studies retrieved from the electronic databases, including the study design, the country where the study was conducted, which research question(s) of interest in this review each study addresses, and the main findings.

#### Study Methods

Of the 63 included studies, the majority adopted a quantitative approach (79.3%). Over half of these studies employed a cross-sectional design (60.3%), most often using questionnaires and surveys, or retrospectively analyzing data sets from hospital admissions or refugee camps. Nineteen studies (30%) were case studies evaluating a specific subset of the refugee population (pregnant women, older populations, etc.). Only 5 studies (7.9%) used a case-control design, comparing the refugee population against a local population. Only one study used a cohort design, and there were none that employed randomized control trial design.

#### Country and Setting

The examination of country distribution of the included studies indicated a similar number of studies on each of Turkey (31.7%), Jordan (26.9%), and Lebanon (26.9%). Very low number of studies was conducted in Syria (6.3%), and Iraq (3.1%). The majority of the data was generated from health centers-based studies (33.3%) such as a hospitals or clinics, while 28.5% of the studies collected data in refugee camps or informal tented settlements.

#### Risk of Bias Assessment

Additional file 6 shows the MMAT risk of bias for all of the 63 included studies. Of the 50 quantitative studies, 28 and 62% were mid- to high-quality (3/4 stars), and high-quality (4/4 stars); respectively. Of the 10 qualitative studies, 50% were of mid- to high-quality (3/4 to 4/4 stars).

#### Thematic Findings

Findings are reported narratively, and are broadly split into “demand side”, addressing health needs; and “supply side”, including gaps in health services, health training, and the health workforce. Results are presented by country.

#### Demand side: health needs

**Fig. 2:**
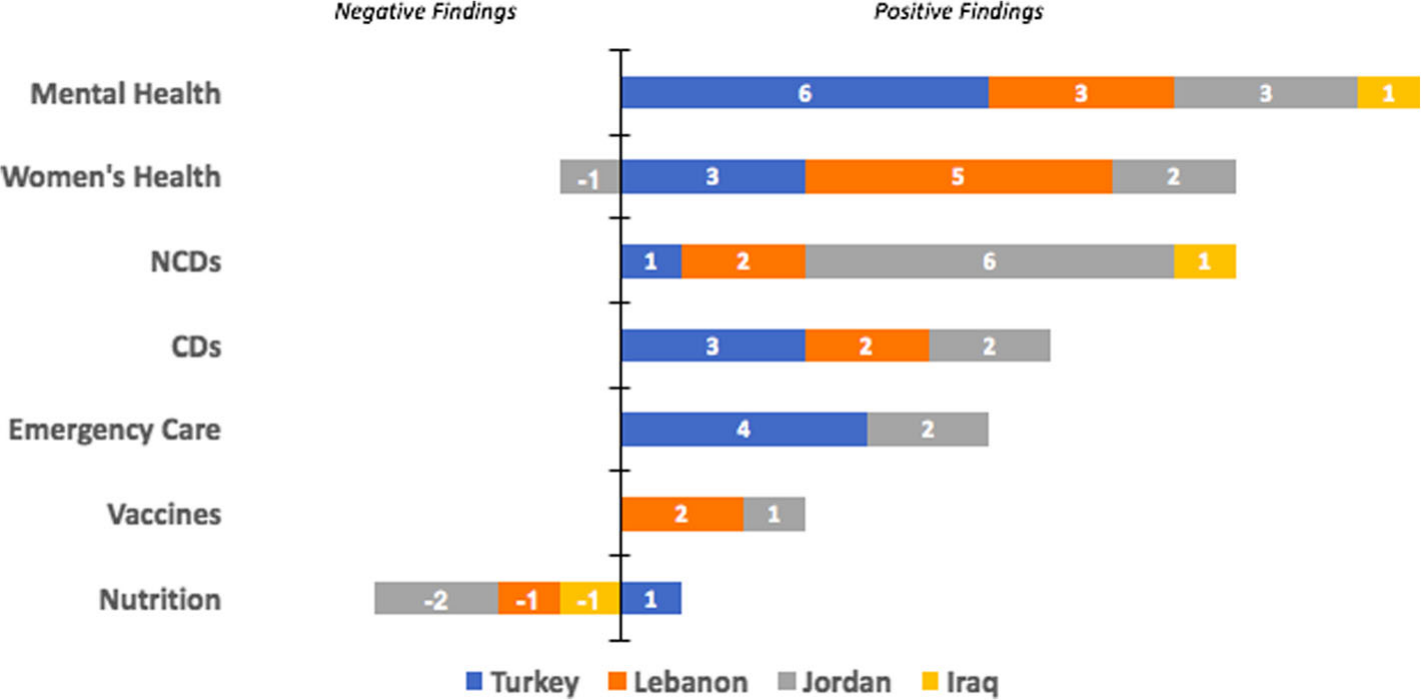
Number of articles addressing health needs of displaced in Syria and its neighboring countries by thematic health group and country (positive findings reflect that there is a need in the health areas presented while negative findings indicate that there isn’t considerable need among the studied population for the services indicated)

The overall number of studies addressing the health needs of Syrians displaced in Syria and to its neighboring countries are represented by country in Fig. 2.

##### Iraq

Only 3 studies tackled health needs in Iraq. One study focused on PTSD among adult Syrian refugees indicating a prevalence rate of 35–38% with no significant gender differences in the occurrence of PTSD [18]. Another study addressed hypertension and musculoskeletal diseases showed rising prevalence rates among higher age groups. The prevalence of hypertension increased with age, with rates of 4.4, 23.9, and 32.1% recorded among age groups of 30–44, 45–59 and 60 and above; respectively [19]. A similar trend was noted for musculoskeletal conditions which prevalence rates increased from 4.2% among those aged 30–44, to 22.4% among those aged 60 and above [19]. Among the surveyed households of this study, 19.4, 13.5 and 9.7% households had at least one member who suffers from hypertension, musculoskeletal conditions, or diabetes; respectively [19].

##### Jordan

Fifteen studies relating to health needs were conducted in Jordan. NCDs [3, 20–24]; women’s health [25–27], mental health [24, 28, 29]; CDs [22, 23], emergency care [22, 30], nutrition [31, 32]; and vaccination [33] were among the studied topics in Jordan. NCDs were reported among the most common health problem in Jordan. Among NCDs, hypertension was found to be highly prevalent, with a rate of 11% reported by Doocy et al. among adult household members [3] and rates of 30 and 41% reported by Al-Fahoum et al. among women and men; respectively [20]. Cardiovascular diseases (CVD) prevalence was noted to be 16% from Gammoh [23] and 23% from Collins et al. [21]. Other reported NCDs included asthma in women (30%) [20], as well as arthritis (7%) and diabetes (6%) across both genders [3]. Studies evaluating women’s health were exclusively related to pregnancies and pregnancy related complications [25–27]. These were mainly focused on the significant rates of anemia (51%), chronic malnutrition (19%), and cesarean deliveries (37%) among Syrian pregnant women. As for studies on mental health, articles focused on psychological distress found that it affects 56% of refugees, while the prevalence rate for PTSD and depression was 18 and 30%, respectively [24, 28, 29].

##### Lebanon

Fifteen studies relating to health needs of Syrian refugees were conducted in Lebanon. Women’s health [27, 34–37] was recorded as the most prevalent health need among Syrians displaced to Lebanon, followed by mental health [4, 38, 39], CDs [7, 40] and vaccinations [33, 41], and finally NCDs [2, 42].

Regarding women’s health, studies were mostly related to the high number of pregnancies and associated complications. Huster et al. [35] found that 44% of all hospital admissions were for delivery. Benage et al. [34] found that only 41% of women were receiving an adequate diet of vitamins during pregnancy. Caesarean sections were reported to be 35% - well above the WHO recommended rate of 5–15% [35]. Moreover, birth complications and infant congenital malformations among Syrian refugees were much higher than the Lebanese population [36]. Lastly, a study from Reese Masterson et al. [37] found a high prevalence of poor reproductive health among displaced Syrian women, including menstrual irregularities (54%), pelvic pain (52%), and reproductive tract infections (53%).

Studies relating to mental health were varied, and assessed PTSD [4], Major Depressive Disorder (MDD) [39], and other disorders, such as schizophrenia and bipolar [38]. Prevalence for all disorders was found to be quite high, with 44% for MDD [39], 38% for schizophrenia [38], and 27% for PTSD [4].

Studies relating to CDs exclusively examined cutaneous leishmaniasis, which was highly specific and becoming increasingly prevalent among the displaced Syrian population [7, 40]. Regarding vaccines, coverage was determined to be insufficient in two studies and was noted remarkably lower than vaccine coverage for the Lebanese population by the two studies [33, 41].

Two studies evaluated NCDs, with high prevalence of hypertension (21%), CVD (11%), and diabetes (10%) reported among the general refugee population [35], and extremely higher prevalence of hypertension (60%), CVD (30%), and diabetes (47%) among refugees aged 60 and above [42].

##### Turkey

Eighteen studies relating to health needs were conducted in Turkey, mainly addressing mental health [5, 43–47]; emergency care [48–51]; women’s health [52–54], and CDs [6, 55, 56].

Regarding mental health, PTSD and depression were found remarkably prevalent [5, 43, 44, 46]. Moreover, Jefee-Bahloul et al. [47] found the refugee population to have high levels of stress scores (42%), and a study [45] researching emotional distress among children, found them to be highly anxious (62%) and fearful (49%).

Possibly due to its proximity to conflicts along the Syrian border, several studies highlighted the need for emergency care among Syrian refugees in Turkey when compared to other countries in the region. A study evaluating hospital admissions found the emergency department to be the most commonly used department [57], and gunshot wounds were found to be the most common cause of hospital admission [51], representing 84% [50] and 70% [5] of all admissions of Syrian refugees to the emergency department.

Similar to Jordan and Lebanon, studies pertaining to women’s health mainly highlighted the high number of pregnancies [53], and high prevalence of caesarean sections (43%) [52]. Studies focusing on CDs mainly examined cutaneous leishmaniasis, which was mostly specific to the Syrian population [6, 56]. A study evaluating chronic malnutrition in youth found the prevalence to be higher than that of Jordan and Lebanon at 19%, and well-above the WHO threshold of 5% [58].

#### Supply side: gaps in services, training, and workforce

##### Health Services

**Fig. 3:**
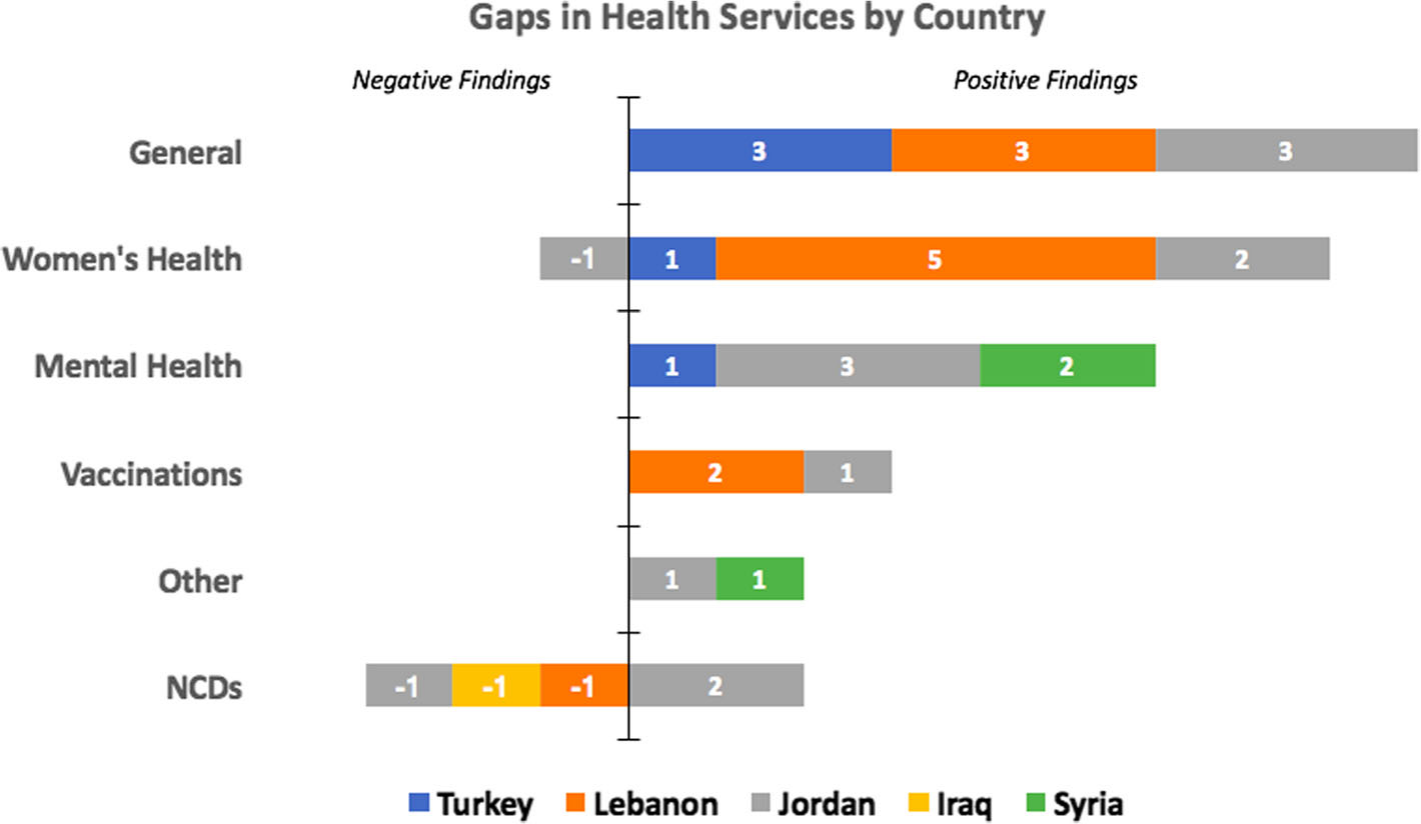
Number of articles addressing gaps in health services provided to Syrians displaced in Syria and its neighboring countries by thematic health group and country (positive findings reflect that there is a gap in the health services corresponding to the health areas presented while negative findings indicate that services provided are sufficient and no gaps exist)

The overall number of studies addressing the gaps in health services provided to Syrians displaced in Syria and its neighboring countries are represented by country in Fig. 3.

##### Iraq

Only one study addressing health service delivery was conducted in Iraq, indicating good access to hypertension, diabetes, and CVD care [19].

##### Jordan

Results from Jordan are summarized in Table 1, with 12 studies relating to gaps in health services. The majority of the studies from Jordan indicated remarkable insufficiency in the availability of services targeting mental health [29] and psychological support [60], general health services [20, 29], as well as health services specific to acute diseases, dental care, vaccinations, and chronic diseases [59]. A gap in women’s health services was also reported, including limited access to quality of obstetrics services [61], clinical management for rape victims [61], and family planning services [63]. Barriers to access reported in literature included cost [27], and lack of adequate knowledge about the importance of seeking necessary services [63]. As for NCDs, gaps in receiving adequate services were mostly due to cost [3] or inability to get an appointment [62].

**Table 1:** Health Service Gaps in Jordan

Author	General Area	Summary of Findings
Ay et al. [59]	General	Needs assessment among PHC services; acute diseases (57%), dental (40%), vaccinations (40%), chronic diseases (37%), OBGYN (33%), emergency care (25%).
Doocy et al. [22]	General	Main barriers to not seeking health care were cost (65%), and medicine being out of stock (10%).
Al-Fahoum et al. [20]	General, Nutrition	Perceived insufficient health services (75%), concerns regarding access to nutrition (64%).
Basheti et al. [29]	General, Mental Health	Disagreed proper medical care was being provided (38%), reported high need for psychological support (46%).
Abo-Hilal et al. [60]	Mental Health	General lack of mental health services – NGO perspective.
Bouchghoul et al. [26]	Women’s Health	Women with antenatal complications successfully referred to a hospital (88%).
Krause et al. [61]	Women’s Health	Limited access to clinical management of rape, quality of obstetric care was criticized by refugees.
Tappis et al. [27]	Women’s Health	Cost was cited as a major factor for care-seeking decisions related to maternal health
Doocy et al. [3]	NCDs	High care seeking behavior for NCD treatment (85%), main barrier was cost.
Al Qadire et al. [62]	NCDs (Cancer)	Gap in seeking medical care for cancer symptoms; did not have insurance (83%), worried what might be found (77%), could not get an appointment (52%).
West et al. [63]	Family Planning	Poor knowledge of available services, low prioritization, need for more female staff.
Roberton et al. [33]	Vaccines	Difficulty in obtaining vaccines (34%).

##### Lebanon

Results of the 11 studies relating to gaps in health services provided to Syrian refugees in Lebanon are summarized in Table 2. General barriers to access included the complexity of the healthcare system in Lebanon [65], cost [42], prejudice from healthcare providers [65], and perceived low interest and concern from the end of the providers [64]. Regarding women’s health, a low number of antenatal care attendance from Syrian women was found to be more prevalent among unregistered women, speculated to be due to cost and lack of accessibility [34, 35]. Generally, the main barrier for both delivery services and access to reproductive health services was reported to be the cost, followed by lack of availability and inaccessibility of reproductive health services [27, 37]. Access to contraceptives [66] is also hindered by cost, as well as unawareness of the services, and refusal from the husband. Gap in vaccine coverage was also reported, with 40% of households reporting difficulties in obtaining vaccinations [33], and only 54% children fully vaccinated [41].

**Table 2:** Health Service Gaps in Lebanon

Author	General Area	Summary of Findings
Doocy et al. [64]	General	Perceived low interest on the provider side; did not ask general questions (60%) or about medication complications (50%).
Parkinson et al. [65]	General	Could not access healthcare due to complex layout, subject to prejudice at hospitals.
Strong et al. [42]	General (Older population)	Cost was the primary reason for not seeking healthcare (79%).
Benage et al. [34]	Women’s Health	Low number of Syrian refugee women had received all 4 minimum antenatal visits compared to Lebanese women.
Cherri et al. [66]	Women’s Health	Main barrier to accessing contraceptives was cost, being unaware of the services, and refusal form the husband.
Huster et al. [35]	Women’s Health	Low antenatal care attendance reported by Syrian women.
Masterson et al. [37]	Women’s Health	Perception that reproductive health services are unavailable (45%), inaccessible (40%); main barrier is cost (50%).
Tappis et al. [27]	Women’s Health	Cost was cited as the primary reason for not seeking delivery location.
Roberton et al. [33]	Vaccines	Difficulty obtaining vaccines (40%).
Rossi et al. [41]	Vaccines	Low prevalence of fully vaccinated children (54%).
Doocy et al. [2]	NCDs	Syrian refugees receive NCD care access at the same frequency as Lebanese host community members

##### Syria

Only 3 studies on gaps in health services in Syria were found. These mainly highlighted the gaps in mental health services, due to severe lack of mental health professionals in the country [67], and severe destruction of mental health service infrastructure [68]. A low number of hemodialysis centers currently operating in the country (52%) was also reported [69].

##### Turkey

Results of the 5 studies relating to gaps in health services provided to Syrian refugees in Turkey are summarized in Table 3. These studies mainly highlighted gaps in mental health services [47], psychosocial services [70], and child psychology services [71]. A survey administered among Turkish healthcare professionals [51] found intensive care (66%) and in-patient care (65%) to be the greatest health service needs. A low number of antenatal care visits among Syrian women was also reported, with 41% of refugee women having had no antenatal care visits throughout their pregnancy.

**Table 3:** Health Service Gaps in Turkey

Author	General Area	Summary of Findings
Savas et al. [51]	General	Highest need cited among healthcare professionals was intensive care capacity (66%) and in-patient care (65%).
Sevinc et al. [70]	General, Mental Health	Difficulty implementing treatments for bureaucratic reasons, lack of psychosocial services.
Sahlool et al. [71]	General, Mental Health	Number of refugee cases seen each day exceeds recommended limit, insufficient rehabilitation and child psychology services.
Jefee-Bahloul et al. [47]	Mental Health	High need for mental health services (34% expressed need to see a psychologist or psychiatrist).
Erenel et al. [53]	Women’s Health	Low level of antenatal care, 41% had no antenatal visits prior to birth.

##### Health Training

*A* total of 9 studies addressing gaps in health training among healthcare professionals were identified in the countries of interest to this paper.

Three studies conducted in Turkey [51, 70, 71] highlighted the need to train Turkish health professionals on communication skills, particularly on speaking Arabic. The 2 studies from Jordan, emphasized on the need to train volunteers on basic skills for assessing and referring psychological problems [60] and the need to train health workers on proper identification and reporting of CVD risk scores [21]. The 2 studies conducted in Lebanon, underlined the need to train field workers on delivering appropriate psychological first aid [72], and the need to develop self-help and mental well-being training programs for field workers [73]. Finally, the 2 studies conducted in Syria underscored the need for higher-level training on advanced mental health skills that go beyond psychological first aid [67], and the need for training technicians on the proper operation of dialysis machines [69].

##### Health Resources

There was a total of 7 studies addressing gaps in health care workers in the countries investigated in this study, with the majority being conducted in Syria (5 out of 7 studies).

Studies from Syria highlighted the lack of healthcare workers and professionals in general [67, 74], with an emphasis on the lack of psychologists [60, 68], and nephrologists [69]. All studies attributed these deficiencies to the purposeful targeting of healthcare workers in Syria forcing them to flee the country. In Lebanon, a general lack of female health care workers was reported [35]. In Turkey, a high level of turn-over among healthcare workers, mainly due to resignation was noted, in addition to overwork was reported, namely due to the considerable increase in health needs with the influx of Syrian refugees to Turkey [51].

##### Grey literature

A summary of findings from the grey literature is presented in Table 4. The grey literature search served to support findings from the published literature; to yield findings from countries where it may be difficult to implement a study (such as Syria), and to gain an up to date picture on the current health needs and service gaps among refugee populations in the region.

**Table 4:** Results from the grey literature

Author	Country	Summary of Findings
UNHCR [75]	Iraq	9% of all households had at least one member living with a disability; 99% reported this member had difficulties accessing services, 93% reported no assistance from an organization
Amnesty International [76]	Jordan	High level of mental health needs, also need to address vulnerable populations.
MSF [77]	Jordan	Thousands denied access to essential medical care – 75% are women and children.
MSF [78]	Jordan	Complicated war injuries persist, long wait lists.
MSF [79]	Jordan	Opening of a mother and child hospital to address gaps in maternal and newborn health.
MSF [80]	Jordan	Difficult and expensive to find treatment for chronic diseases for those living outside of camps.
UNHCR [81]	Jordan	Need for more female health workers, more reproductive health services for men, and more mental health support.
Amnesty International [82]	Lebanon	General lack of secondary and tertiary care. High treatment costs for cancer and NCDs.
ICRC [83]	Lebanon	Increase in the number of wounded patients and shelter priorities due to winter.
ICRC [84]	Lebanon	War-related surgery procedures are extremely prevalent.
UNHCR [85]	Lebanon	Chronic illnesses were the primary health need across all governorates; 16% could not access healthcare - 93% of those due to cost.
MSF [86]	Syria	Raqqa: Major difficulties obtaining urgent lifesaving medical care due to ongoing battles.
MSF [87]	Syria	Aleppo: Significant increases in the number of wounded patients.
MSF [88]	Syria	Low vaccination rates and potential measles outbreaks.
MSF [89]	Syria	Continued understaffing and funding of medical facilities; gaps in mental health, vaccines, chronic diseases, reproductive health, and secondary and tertiary care.
MSF [90]	Syria	Food shortages, lack of good nutrition. Accessibility to maternal hospitals is limited.
MSF [91]	Syria	Shortage of doctors in Aleppo due to targeted airstrikes.
MSF [92]	Syria	Many children currently unvaccinated, undocumented cases of measles, meningitis, and pneumonia.
UNICEF [93]	Syria	Among youth, there is a high prevalence of malnutrition and malnourishment, re-emergence of polio, severe psychological problems.
UNHCR [94]	Turkey, Jordan, and Iraq	*Iraq*: Vaccination campaigns being provided. *Jordan*: Support of reproductive health services. *Turkey*: Hygiene kits being delivered to communities.
WHO [95]	Iraq, Jordan, Lebanon, Syria, and Turkey	*Iraq*: Measles outbreak, upper respiratory tract infections. *Jordan*: War-related injuries. *Lebanon*: Maternal and child health services as priority need amongst Syrian refugees in Lebanon, along with mental health and NCD services. *Syria*: Vulnerable to infectious disease outbreaks, acute jaundice syndrome, and typhoid. *Turkey*: CDs, vaccine-preventable diseases, and mental health
WHO [96]	Lebanon, Jordan, and Iraq	*Mental Health*: 50% estimated to be in need of psychosocial support. *Reproductive, maternal, and child health*: Low use of antenatal care, high rates of caesarean sections. *NCDs*: High prevalence in Syria and Jordan. *CDs*: Outbreak due to migration. Injuries also a high priority.

##### Iraq

A UNHCR report on refugees living with disabilities, found an extreme gap in services being provided for this population, including difficulty accessing services, and lack of assistance from any organization. On a separate note, significant measles outbreaks, as well as upper-respiratory tract infections were reported among refugee populations [95]. A regional report from the WHO emphasized gaps in psychosocial support, as well low use of antenatal care and high rates of caesarean sections [96].

##### Jordan

The grey literature from Jordan highlighted a variety of health needs including a high number of complicated injuries due to war, and a corresponding lack of services being provided [95]. Amnesty International pointed to the high level of mental health needs, especially among vulnerable populations [76]. MSF reported on the high number of women and children who are currently being denied access to medical care [77], and on the difficulty and cost associated with finding treatment for chronic diseases [80]. UNHCR underlined the need for more female healthcare workers, reproductive health services, and mental health support [81, 94] with a news update from MSF [79] further underscoring these needs.

##### Lebanon

Reports generated by the ICRC, found war-related injuries in Lebanon to be extremely prevalent [83, 84]. NCDs were also found to be highly prevalent, and were the most common health need across all governorates [85]; however, treatment costs for NCDs were also found to be extremely high [82].

##### Syria

Of the grey literature relating to Syria, reports outlined all the expected health needs and service gaps associated with conflict zones. These included: shortages of doctors [91] and understaffing of medical facilities [89]; difficulties in receiving care due to ongoing battles [86], as well as increase in the number of wounded patients [87]; low vaccination rates, with undocumented outbreaks of measles, meningitis, and pneumonia [88, 92]; and finally, food shortages [90], with a high prevalence of malnutrition and malnourishment [93].

### Validation of Lebanon-Specific Results

Additional file 7 presents the results of the validation meeting conducted with the directors and representatives of MOPH PHCs with regards to prioritizing the health needs. From a practical point of view, the majority of the MOPH PHCs representatives indicated that CDs are one of the top priority health needs among refugees. More specifically, MOPH PHCs representatives elaborated on the need for increased vaccination, as it remains a challenge with children who constitute the majority of displaced Syrians in Lebanon. Scabies and Leishmania were among the most encountered cases of CDs. Of similar importance is women’s health, where MOPH PHCs representatives indicated that a high number of PHC visits are related to pregnancy complications. The representatives also highlighted the lack of knowledge and awareness on the importance of antenatal care. Mental health was also perceived as a top priority health need among refugees by MOPH PHCs, which indicated that pediatric psychosocial support remains highly needed in light of the prevalent child abuse and depression cases among children and adolescents. NCDs were thought of as the least urgent health need to address, given the integrated NCD program implemented by the MOPH at the level of PHCs.

In parallel, roundtable discussions with NGOs and humanitarian aid agencies representatives yielded similar results, with women’s health, mental health, and CDs reported as the main health needs of Syrian refugees residing in Lebanon. At the level of women’s health, adequate awareness on safe motherhood practices, symptoms of complications during pregnancy, and family planning was thought to be lacking. Early marriage was flagged out as one of the main encountered predictors for maternal and neonatal morbidity and mortality. Enhancing access to antenatal care and postnatal care services was perceived as necessary. As for mental health, representatives stressed on the importance of destigmatizing mental health services. They also underlined the need for more effective community outreach, detection and referral system, as well as synchronized psychosocial support and counselling services among different NGOs and aid agencies. With regards to CDs, a more solid surveillance and referral system in refugee settings was deemed necessary to detect emergent cases of CDs such as polio and mitigate potential outbreaks. Also under the umbrella of CDs, general lack of awareness on the importance of vaccination was also noted, which hinders reaching ultimate rates of vaccination coverage.

Representatives also stated overarching barriers impeding access to adequate services by refugees, which exacerbates the severity of health needs. These include geographical barriers such as transportation and cost of services, and more importantly, lack of necessary awareness and education about the diseases’ symptoms, treatment, and corresponding available services. The roundtable discussion also featured the need to harmonize the work of different NGOs and agencies to respond to the refugee crisis, and prevent duplication of effort.

## Discussion

This study is the first to systematically review the health needs of Syrians displaced in Syria and its neighboring countries including Lebanon, Jordan, Turkey, and Iraq. Regarding the health needs defined as health problems that can be addressed by direct delivery of health services, the prevalence of mental and women’s health needs in Jordan, Lebanon, and Turkey were remarkably high after compiling the existing literature. Specifically, mental health studies found high rates of PTSD and depression. Women’s health studies found high rates of antenatal and pregnancy-related complications. CDs were highly cited; however, this mostly referred to outbreaks of cutaneous leishmaniasis among refugee populations in Lebanon and Turkey. Studies from Turkey specifically emphasized the high level of emergency care needs, including emergency department visits and gunshot wounds, which were also prevalent in Jordan but to a lesser degree. Additionally, while there were few studies researching vaccination coverage, it was found to be insufficient in both Lebanon and Jordan. Finally, nutrition-related health concerns, such as mal-nutrition and mal-nourishment, were found to be acceptable as per WHO standards in Iraq, Jordan, and Lebanon; but unacceptable in Turkey.

An evident gap in health services currently provided to Syrian refugees in Jordan, Lebanon, and Turkey was highlighted through the results. However, the nature of these services differed between countries. In Jordan, gaps were heterogeneous, and related to women’s health, mental health, and vaccine coverage, with conflicting findings regarding NCD services. In Lebanon, this gap was mostly within women’s health, and included insufficient antenatal visits, and other delivery services. Studies further highlighted logistical and societal gaps, including the complex nature of humanitarian health services, and the prejudice experienced by refugees from service providers. Regarding Turkey, gaps were mostly related to mental health. Notably, while NCDs were found to be a highly prevalent health need, most studies indicated that health services have been keeping up with this demand. Due to the low yield of studies relating to the research questions, on the availability of skilled human resources and the gap in training, it remains challenging to make general conclusions with regards to these two themes.

The grey literature essentially supported our findings from the electronic databases search, while also broadening findings from Syria. While there were a number of topics touched upon, there are a few main conclusions. Namely, most reports emphasized the considerable number of vulnerable populations requiring healthcare in the region, including women, children, elderly, and those living with disability. Additionally, the grey literature reiterated the low vaccination rates across Jordan, Lebanon, and Syria, as well as outbreaks of vaccine-preventable diseases, such as measles. Finally, a major shortage across all health services and healthcare workers within Syria was noted.

A dearth of studies conducted in Iraq was noted leading consequently to few conclusions made about this country. While Iraq hosts a significantly less number of refugees than Jordan, Lebanon, and Turkey, the country still host an estimated 250,000 Syrians – no small amount [1]. Therefore, more effort should be invested to understand the health needs of Syrian refugees in Iraq.

### Strengths and Limitations

To the best of our knowledge, this is the first study to systematically review the health needs and service gaps among displaced Syrians in the refugee-hosting countries neighboring Syria. The study also allocated a unique emphasis on the health needs of refugees in Lebanon. The electronic databases search yielded a high number of studies, which allowed for a detailed analysis. The review of the grey literature complemented evidence generated from the latter and generated more recent results, as well as more elaborate information pertaining to Syria.

However, there remain a number of limitations pertaining to our study. Due to the nature of the research questions, most of the studies included in the analysis were quantitative, cross-sectional studies, and therefore, more susceptible to bias. However, using the MMAT, 70% of studies were found to be of higher quality, thereby accounting for this limitation. Despite this, the results of our review could not be synthesized into a meta-analysis, and consequently, more specific conclusions, such as pooled prevalence rates, could not be made. Also, despite the importance of the grey literature in complementing results obtained from the electronic databases, the search of grey literature was not comprehensive and may have missed the portals of several international organizations significantly involved in the response to the health needs of Syrian refugees in Lebanon. Additionally, discussions of the meeting conducted to validate the Lebanon-specific results of the systematic review may have encompassed potential biases such as the perception of the representatives of the definition of health needs. While the term health needs was defined at the beginning of each of the meetings, the actual understanding and description of the term may differ from one individual to another. In other words, the term health needs could have been perceived by the representatives as the cases that health providers are unable to manage adequately due to the lack of skills needed, or those to which shortage of effective health services is noted, as a result of unavailability of necessary infrastructure, instead of merely prevalence of cases. Also, the representatives of the MOPH PHCs reflected on their experience and practice in their respective PHCs, which are located in six different regions in Lebanon. While this choice of variety of locations represented was deliberate, it may have resulted in a more challenging ability to reach consensus among the group, due to the difference in health needs across different communities of refugees served in these different locations.

### Implications

Moving towards a more coherent and synchronized response to the health needs of refugees in Lebanon and the region remains necessary with the protracted aspect of the crisis in Syria. This is of particular importance in lights of the financial fatigue faced by the different actors trying to alleviate the burden placed on the health systems of the refugee hosting countries. Results of this comprehensive review constitute therefore a valuable, inclusive, and evidence-based reference for international and local policymakers and stakeholders to effectively address the current refugee crisis. It can additionally assist NGOs, humanitarian aid agencies, and other actors to make decisions related to investments and allocation of budgets to interventions targeting the priority health needs of Syrian refugees concluded in this study. The presentation of the results by country may also assist those actors in conducting more targeted planning for interventions at the level of each country. On the other hand, the highlighted gaps in health training and human resources provides baseline data that could guide capacity building initiatives in each of the studied countries.

## Conclusion

An enhanced synchronized approach to address the priority health needs of Syrian refugees in Lebanon and Syria’s neighboring countries remains highly needed to reduce the existing gaps identified in responding to the health needs of displaced Syrians. This includes training of health human resources to ensure a skilled workforce, adequate infrastructure for the provision of health services needed, and community-based efforts to overcome barriers to access. In Lebanon specifically, women’s health, mental health, and communicable diseases remain the most prevalent health needs among the Syrian refugee population. Having said that, service interventions and capacity building initiatives in these health areas are highly encouraged.

## Additional files

Additional file 1: **Appendix 1.** Results of the searches of the electronic databases. (DOCX 40 kb)

Additional file 2: **Appendix 2.** Search strategies for the published literature. (DOCX 143 kb)

Additional file 3: **Appendix 3.** Search strategies for grey literature (DOCX 85 kb)

Additional file 4: **Table S1.** Excluded studies with reasons for exclusions from the database search. (DOCX 171 kb)

Additional file 5: **Table S2.** Summary of studies included for analysis from the database search. (DOCX 101 kb)

Additional file 6: **Table S3**. MMAT Risk of Bias Summary. (DOCX 110 kb)

Additional file 7: Results of the validation meeting on health needs prioritization, conducted with the directors and representatives of MOPH PHCs. (PNG 39 kb)

## Data Availability

All data related to this study are included in this published article and its supplementary information files.

## Abbreviations

CD: Communicable diseases
CINAHL: Cumulative Index to Nursing and Allied Health Literature
CVD: Cardiovascular disease
EMRO: WHO Eastern Mediterranean Regional Office
ICRC: International Committee of the Red Cross
MDD: Major Depressive Disorder
MMAT: McGill Mixed Methods Appraisal
MOPH: Ministry of Public Health
MSF: Médecins Sans Frontières
NCD: Non-communicable diseases
NGO: Non-governmental organization
PHC: Primary healthcare centre
PRISMA: Preferred Reporting Items for Systematic Reviews and Meta-Analyses
PTSD: Post-traumatic stress disorder
UNHCR: United Nations High Commissioner for Human Rights
WHO: World Health Organization
