# Supplementary material for: Assessment of the health needs of Syrian refugees in Lebanon and Syria’s neighboring countries
Source: Confl Health. 2019 Jun 27;13:31. doi: 10.1186/s13031-019-0211-3 (PMC6598365; doi:10.1186/s13031-019-0211-3)
Supplement: Supplementary file 1 — Appendix 1. Results of the searches of the electronic databases. (DOCX 40 kb) [file 13031_2019_211_MOESM1_ESM.docx]

# Appendix 1: Results of the searches of the electronic databases

| **Database** | **Number of Hits** | **Date of Search** |
| --- | --- | --- |
| Medline | 3 678 | 16/08/2017 |
| PubMed | 1 259 | 16/08/2017 |
| EMBASE | 5 474 | 16/08/2017 |
| Scopus | 2 974 | 16/08/2017 |
| CINAHL | 1 215 | 16/08/2017 |
| Global Health Library | 3 657 | 16/08/2017 |
