# Supplementary material for: Assessment of the health needs of Syrian refugees in Lebanon and Syria’s neighboring countries
Source: Confl Health. 2019 Jun 27;13:31. doi: 10.1186/s13031-019-0211-3 (PMC6598365; doi:10.1186/s13031-019-0211-3)
Supplement: Supplementary file 2 — Appendix 2. Search strategies for the published literature. (DOCX 143 kb) [file 13031_2019_211_MOESM2_ESM.docx]

**Appendix 2:** Search strategies for the published literature

*Medline Search Strategy*

1 exp education, dental/ or exp education, medical/ or exp education, nursing/ or education, public health professional/ or exp health status/ or health transition/ or exp vital statistics/ or exp health/ or capacity building/ or health communication/ or exp health facilities/ or health manpower/ or exp health personnel/ or exp health promotion/ or exp health services/ or health priorities/ or health resources/ or exp health care surveys/ or "health services needs and demand"/ or needs assessment/ or "catchment area (health)"/ or medically underserved area/ or regional medical programs/ or healthcare disparities/ or practice patterns, dentists'/ or practice patterns, nurses'/ or practice patterns, physicians'/ or professional practice gaps/ or community-based participatory research/ or public health systems research/ or clinical competence/ or disease eradication/ or exp disease outbreaks/ or emergencies/ or endemic diseases/ or exp public health practice/ (4,160,664)

2 (health* adj3 (access* or status* or transition? or level? or need? or demand? or priorit* or gap? or service? or problem? or resource? Or utili?ation?)).mp. [mp=title, abstract, original title, name of substance word, subject heading word, keyword heading word, protocol supplementary concept word, rare disease supplementary concept word, unique identifier, synonyms] (700,945)

3 (((health* or medical) adj (worker? or professional? or provider? or manpower or student? or trainee? or intern? or resident? or graduate?)) or medic? or paramedic? or doctor? or practitioner? or physician? or clinician? or nurse? or pharmacist? or dentist? or dieti?ian? or nutritionist? or physical therapist? or psycho?social or psychiatrist? or psychologist?).mp. [mp=title, abstract, original title, name of substance word, subject heading word, keyword heading word, protocol supplementary concept word, rare disease supplementary concept word, unique identifier, synonyms] (1,178,302)

4 (health* adj2 (practice? or survey? or training or education or (capacity adj building) or promotion or development or assessment? or outcome? or impact)).mp. [mp=title, abstract, original title, name of substance word, subject heading word, keyword heading word, protocol supplementary concept word, rare disease supplementary concept word, unique identifier, synonyms] (454,069)

5 ((disease? or endemic? or pandemic? or pathogen?) adj5 (status* or level? or outbreak? or out-break? or transmission? or prevalence or incidence or eliminat* or eradicat*)).mp. [mp=title, abstract, original title, name of substance word, subject heading word, keyword heading word, protocol supplementary concept word, rare disease supplementary concept word, unique identifier, synonyms] (278,203)

6 or/1-5 (4,990,654)

7 disaster victims/ or exp "emigrants and immigrants"/ or refugees/ or "transients and migrants"/ or vulnerable populations/ (34,034)

8 (refugee? or migrant? or emigrant? or immigrant? or transient? or nomad? or ((displaced or relocat* or re-locat* or vulnerable) adj2 (individual? or person? or people or famil* or nation? or communit* or population?)) or (asylum adj2 seeker?) or exodus).mp. [mp=title, abstract, original title, name of substance word, subject heading word, keyword heading word, protocol supplementary concept word, rare disease supplementary concept word, unique identifier, synonyms] (342,457)

9 ((war* or forced or conflict? or (armed adj conflict) or disaster?) adj3 (victim? or survivo?r?)).mp. [mp=title, abstract, original title, name of substance word, subject heading word, keyword heading word, protocol supplementary concept word, rare disease supplementary concept word, unique identifier, synonyms] (1,900)

10 or/7-9 (344,208)

11 exp middle east/ or iraq/ or jordan/ or lebanon/ or syria/ or turkey/ or arabs/ or (iraq* or jordan* or lebanon or lebanese or libanaise or turkey or Syria* or EMRO or ((middle or near) adj2 east*) or (east* adj2 mediterranean) or orient or arabs or arab or arabia or levant).mp. [mp=title, abstract, original title, name of substance word, subject heading word, keyword heading word, protocol supplementary concept word, rare disease supplementary concept word, unique identifier, synonyms] (172,368)

12 6 and 10 and 11 (3,574)

-------------------------------------------

*PubMed Search Strategy*

1 education, dental [mesh] OR education, medical [mesh] OR education, nursing [mesh] OR education, public health professional [mesh] OR health status [mesh] OR health transition [mesh] OR vital statistics [mesh] OR health [mesh] OR capacity building [mesh] or health communication [mesh] OR health facilities [mesh] OR health manpower [mesh] OR health personnel [mesh] OR health promotion [mesh] OR health services [mesh] OR health priorities [mesh] OR health resources [mesh] OR health care surveys [mesh] OR health services needs and demand [mesh] OR healthcare disparities [mesh] OR practice patterns, dentists’ [mesh] OR practice patterns, nurses’ [mesh] OR practice patterns, physicians’ [mesh] OR professional practice gaps [mesh] OR community-based participatory research [mesh] OR public health systems research [mesh] OR clinical competence [mesh] OR disease eradication [mesh] OR disease outbreaks [mesh] OR emergencies [mesh] OR endemic disease [mesh] OR public health practice [mesh] (4,012,870)

2 health access* [tw] OR healthcare access* [tw] OR health-care access* [tw] OR health status* [tw] OR healthcare status* [tw] OR health-care status* [tw] OR health transition* [tw] OR healthcare transition* [tw] OR health-care transition* [tw] OR health level* [tw] OR healthcare level* [tw] OR health-care level* [tw] OR health need* [tw] OR healthcare need* [tw] OR health-care need* [tw] OR health demand* [tw] OR healthcare demand* [tw] OR health-care demand* [tw] OR health priorit* [tw] OR healthcare priorit* [tw] OR health-care priorit* [tw] OR health gap* [tw] OR healthcare gap* [tw] OR health-care gap* [tw] OR health service* [tw] OR healthcare service* [tw] OR health-care service* [tw] OR health problem* [tw] OR healthcare problem* [tw] OR health-care problem* [tw] OR health resource* [tw] OR healthcare resource* [tw] OR health-care resource* [tw] – (596,169)

3 health worker* [tw] OR healthcare worker* [tw] OR health-care worker* [tw] OR health professional* [tw] OR healthcare professional* [tw] OR health-care professional* [tw] OR health provider* [tw] OR healthcare provider* [tw] OR health-care provider* [tw] OR health manpower* [tw] OR healthcare manpower* [tw] OR health-care manpower* [tw] OR health student* [tw] OR healthcare student* [tw] OR health-care student* [tw] OR health trainee* [tw] OR healthcare trainee* [tw] OR health-care trainee* [tw] OR health intern* [tw] OR healthcare intern* [tw] OR health-care intern* [tw] OR health resident* OR [tw] healthcare resident* [tw] OR health-care resident* [tw] OR health graduate* OR [tw] healthcare gradute* [tw] OR health-care graduate* [tw] OR medical worker* [tw] OR medical worker* [tw] OR medical worker* [tw] OR medical professional* [tw] OR medical professional* [tw] OR medical professional* [tw] OR medical provider* [tw] OR medical provider* [tw] OR medical provider* [tw] OR medical manpower* [tw] OR medical manpower* [tw] OR medical manpower* [tw] OR medical student* [tw] OR medical student* [tw] OR medical student* [tw] OR medical trainee* [tw] OR medical trainee* [tw] OR medical trainee* [tw] OR medical intern* [tw] OR medical intern* [tw] OR medical intern* [tw] OR medical resident* OR [tw] medical resident* [tw] OR medical resident* [tw] OR medical graduate* OR [tw] medical graduate* [tw] OR medical graduate* [tw] OR medic* [tw] OR paramedic* [tw] OR doctor* [tw] or practitioner* [tw] OR physician* [tw] OR clinician* [tw] or nurse* [tw] OR pharmacist* [tw] OR dentist* [tw] OR dietitian* [tw] OR nutritionist* [tw] OR physical therapist* [tw] OR psychosocial [tw] OR psycho-social [tw] OR psychiatrist* [tw] OR psychologist* [tw]– (1,243,832)

4 health practice* [tw] OR healthcare practice* [tw] OR health-care practice* [tw] OR health survey* [tw] OR healthcare survey* [tw] OR health-care survey* [tw] OR health training [tw] OR healthcare training [tw] OR health-care training [tw] OR health education [tw] OR healthcare education [tw] OR health-care education [tw] OR health capacity building [tw] OR healthcare capacity building [tw] OR health-care capacity building [tw] OR promotion [tw] OR healthcare promotion [tw] OR health-care promotion [tw] OR health development [tw] OR healthcare development [tw] OR health-care development [tw] OR health assessment* [tw] OR healthcare assessment* [tw] OR health-care assessment* [tw] – (309,186)

5 disease status* [tw] OR disease level* [tw] OR disease outbreak* [tw] OR disease out-break* [tw] OR disease transmission* [tw] OR disease prevalence [tw] OR disease incidence [tw] OR disease eliminate* [tw] OR disease eradicat* [tw] OR endemic status* [tw] OR endemic level* [tw] OR endemic outbreak* [tw] OR endemic out-break* [tw] OR endemic transmission* [tw] OR endemic prevalence [tw] OR endemic incidence [tw] OR endemic eliminat* [tw] OR endemic eradicat* [tw] OR pandemic status* [tw] OR pandemic level* [tw] OR pandemic outbreak* [tw] OR pandemic out-break* [tw] OR pandemic transmission* [tw] OR pandemic prevalence [tw] OR pandemic incidence [tw] OR pandemic eliminat* [tw] OR pandemic eradicat* [tw] OR pathogen status* [tw] OR pathogen level* [tw] OR pathogen outbreak* [tw] OR pathogen out-break* [tw] OR pathogen transmission* [tw] OR pathogen prevalence [tw] OR pathogen incidence [tw] OR pathogen eliminate* [tw] OR pathogen eradicat* [tw] – (148,391)

6 OR/#1-#5 – (4,750,351)

7 disaster victim [mesh] OR emigrants and immigrants [mesh] OR refugees [mesh] OR transients and migrants [mesh] OR vulnerable populations [mesh] – (32,602)

8 refugee* [tw] OR migrant* [tw] OR emigrant* [tw] OR immigrant* [tw] OR transient* [tw] OR nomad* [tw] OR asylum seeker* [tw] OR displaced individual* [tw] OR displaced person* [tw] OR displaced people [tw] OR displaced famil* [tw] OR displaced nation* [tw] OR displaced communit* [tw] OR displaced population* [tw] OR relocated individual* [tw] OR relocated person* [tw] OR relocated people [tw] OR relocated famil* [tw] OR relocated nation* [tw] OR relocated communit* [tw] OR relocated population* [tw] OR re-located individual* [tw] OR re-located person* [tw] OR re-located people [tw] OR re-located famil* [tw] OR re-located nation* [tw] OR re-located communit* [tw] OR re-located population* [tw] OR vulnerable individual* [tw] OR vulnerable person* [tw] OR vulnerable people [tw] OR vulnerable famil* [tw] OR vulnerable nation* [tw] OR vulnerable communit* [tw] OR vulnerable population* [tw] or exodus [tw] – (372,434)

9 war victim* [tw] OR war survivor* [tw] OR war survivour* [tw] OR warfare victim* [tw] OR warfare survivor* [tw] OR warfare survivour* [tw] OR conflict victim* [tw] OR conflict survivor* [tw] OR conflict survivour* [tw] OR armed conflict victim* [tw] OR armed conflict survivor* [tw] OR armed conflict survivour* [tw] OR disaster victim* [tw] OR disaster survivor* [tw] OR disaster survivour* [tw] – (3,274)

10 OR/7-9 – (375,502)

11 middle east [mesh] OR iraq [mesh] OR jordan [mesh] OR lebanon [mesh] OR Syria [mesh] OR turkey [mesh] OR arabs [mesh] iraq* [tw] OR jordan* [tw] OR lebanon [tw] OR lebanese [tw] OR libanaise [tw] OR turkey [tw] OR syria* [tw] EMRO [tw] OR middle east* [tw] OR east mediterranean [tw] OR eastern mediterranean [tw] OR near east* [tw] OR orient [tw] OR arabs [tw] OR arab [tw] OR arabia [tw] OR levant [tw] – (45,180)

12 #6 AND #10 AND #11 – (1,151)

-------------------------------------------

*Embase Search Strategy*

1 ‘health statistics’/exp OR ‘vital statistics’/exp OR ‘health status’/exp OR ‘mental disease’/exp OR ‘physical disease’/exp OR ‘health care facility’/exp OR ‘health service’/exp OR ‘health care manpower’/exp OR ‘health care utilization’/exp OR ‘health impact assessment’/exp OR ‘health care system’/exp OR ‘national health service’/exp OR ‘non profit organization’/exp OR ‘dental practice’/exp OR ‘health care practice’/exp OR ‘medical practice’/exp OR ‘social work practice’/exp OR ‘public health service’/exp OR ‘health care personnel’/exp OR ‘health care survey’/exp OR ‘health equity’/exp OR ‘practice gap’/exp OR ‘public health systems research’/exp OR ‘doctoral education’/exp or ‘medical education’/exp (21,100,879)

2 (health* NEAR/3 (access* or statistic or statistics or status or level or levels or service or services or gap or gaps or problem or problems or need or needs or demand or demands or priorit* or utili?ation)):ti,ab,kw (415,307)

3 (((health* or medical) NEAR/1 (worker or workers or professional or professionals or provider or providers manpower or student or students or trainee or trainees or intern or interns or resident or residents or graduate or graduates)) or medic or medics or paramedic or paramedics or doctor or doctors or practitioner or practitioners or physician or physicians or nurse or nurses or pharmacist or pharmacists or dentist or dentists or dieti?ian or dieti?ians or psycho?social or psychiatrist or psychiatrists or psychologist or psychologists):ti,ab,kw (999,005)

4 ((health* NEAR/2 (practice OR survey OR training OR education OR promotion OR development OR assessment OR outcome OR outcomes OR impact)) OR (capacity NEAR/1 building))ti,ab,kw (234,299)

5 ((disease or diseases or endemic or endemics or pandemic or pandemics or pathogen or pathogens or illness or abnormalit* or impairment) NEAR/5 (status* or level or levels or outbreak or outbreaks or out-break or out-breaks or transmission or transmissions or prevalence or incidence or eliminat* or eradicat*)):ti,ab,kw (281,944)

6 #1 OR #2 OR #3 OR #4 OR #5 (21,261,074)

7 'refugee'/exp OR 'immigrant'/exp OR 'emigrant'/exp OR 'undocumented immigrant'/exp OR 'vulnerable population'/exp (33,612)

8 (refugee or refugees or migrant or migrants or emigrant or emigrants or immigrant or immigrants or transient or transients or nomad or nomads or ((displaced or relocat* or re-locat* or vulnerable) NEAR/3 (individual or individuals or person or persons or people or peoples or famil* or nation or nations or communit* or population or populations)) or (asylum NEAR/2 (seeker or seekers)) or exodus):ti,ab,kw (367,544)

9 ((war or wars or warfare or conflict or conflicts or ‘armed conflict’ or disaster or disasters) NEAR/3 (victim or victims or survivo?r or survivo?rs)):ti,ab,kw (1,350)

10 #7 OR #8 OR #9 (380,542)

11 'middle east'/exp or ‘iraq’/exp or ‘jordan’/exp or ‘lebanon'/exp OR 'syrian arab republic'/exp or ‘turkey’/exp or 'arab'/exp or (iraq* or jordan* or lebanon or lebanese or libanaise or turkey or Syria* or EMRO or ((middle or near) NEAR/2 east*) or (east* NEXT/2 mediterranean) or orient or arabs or arab or arabia or levant):ti,ab,kw (209,311)

12 #6 AND #10 AND #11 (5,057)

-------------------------------------------

*Scopus Search Strategy*

1 TITLE-ABS-KEY(health* W/3 (access* or status* or transition* or level* or need* or demand* or priorit* or gap* or service* or problem* or resource* or utili?ation)) (1,151,067)

2 TITLE-ABS-KEY(((health* or medical) W/1 (worker* or professional* or provider* or manpower or student* or trainee* or intern* or resident* or graduate*)) or medic* or paramedic* or doctor* or practitioner* or physician* or clinician* or nurse* or pharmacist* or dentist* or dieti?ian* or nutritionist* or physical therapist* or psycho?social or psychiatrist* or psychologist*) (26,980)

3 TITLE-ABS-KEY (( health* W/2 (practice* OR survey* OR training OR education OR "capacity building" OR promotion OR development OR assessment OR outcome* OR impact))) (8001,747)

4 TITLE-ABS-KEY((disease or endemic or pandemic or pathogen) W/5 (status* or level* or outbreak* or out-break* or transmission* or prevalence or incidence or eliminat* or eradicat*)) (428,651)

5 #1 OR #2 OR #3 OR #4 (2,083,449)

6 TITLE-ABS-KEY(refugee* or migrant* or emigrant* or immigrant* or transient* or nomad* or ((displaced or relocat* or re-locat* or vulnerable) W/2 (individual* or person* or people or famil* or nation* or communit* or population*)) or (asylum W/2 seeker*) or exodus) (862,373

7 TITLE-ABS-KEY((war* or forced or conflict or (armed W/1 conflict) or disaster) W/3 (victim or survivo?r)) (3,628)

8 #6 OR #7 (865,756)

9 TITLE-ABS-KEY((middle W/2 east*) OR (east* W/2 mediterranean) OR lebanon OR lebanese OR iraq* OR jordan* OR libanaise OR Syria* OR turkey OR levant OR EMRO OR orient OR arabs OR arab OR arabia) (370,612)

10 #5 AND #8 AND #9 (1,939)

-------------------------------------------

*CINAHL Search Strategy*

1 (MH "Education, Pharmacy") OR (MH "Education, Nursing, Diploma Programs") OR (MH "Education, Nursing, Associate") OR (MH "Social Work+") OR (MH "Social Work, Psychiatric") OR (MH "Vital Statistics+") OR (MH "Health Status+") OR (MH "Health Status Disparities") OR (MH "Health Status Indicators") OR (MH "Health Services Accessibility") OR (MH "Health Services") OR (MH "Health Services Needs and Demand+") OR (MH "Health Services for Persons with Disabilities") OR (MH "Community Mental Health Services+") OR (MH "Adolescent Health Services") OR (MH "Health Manpower") OR (MH "Personnel, Health Facility") OR (MH "Healthcare Disparities") OR (MH "Disease Outbreaks") (360,079)

2 (health* N3 (access* or status* or transition# or level# or need# or demand# or priorit* or gap# or service# or problem# or resource#)) (340,647)

3 (((health* or medical) N1 (worker# or professional# or provider# or manpower or student# or trainee# or intern# or resident# or graduate#)) or medic# or paramedic# or doctor# or practitioner# or physician# or clinician# or nurse# or pharmacist# or dentist# or dieti#ian# or nutritionist# or physical therapist# psycho#social or psychiatrist# or psychologist#) (714,673)

4 (health* N2 (practice# or survey# training or education or “capacity building” or promotion or development or assessment#)) (134,315)

5 ((disease# or endemic# or pandemic# or pathogen#) N5 (status* or level# or outbreak# or out-break# or transmission# or prevalence or incidence or eliminat* or eradicat*)) (58,314)

6 S1 OR S2 OR S3 OR S4 OR S5 (1,259,915)

7 (MH "Refugees") OR (MH "Immigrants+") OR (MH "Immigrants, Illegal") OR (MH "Emigration and Immigration") (19,173)

8 (refugee# or migrant# or emigrant# or immigrant# or transient# or nomad# or ((displaced or relocat* or re-locat* or vulnerable) N2 (individual# or person# or people or famil* or nation# or communit* or population#)) or (asylum N2 seeker#) or exodus) (47,642)

9 ((war* or forced or conflict# or (armed N1 conflict) or disaster#) N3 (victim# or survivo#r#)) (705)

10 S7 OR S8 OR S9 (51,040)

11 ((MH "Middle East+") OR (TI ((middle W2 east*) OR (east* W2 mediterranean) OR lebanon OR lebanese OR iraq* OR jordan* OR libanaise OR Syria* OR turkey OR levant OR EMRO OR orient OR arabs OR arab OR arabia) OR AB ((middle W2 east*) OR (east* W2 mediterranean) OR lebanon OR lebanese OR iraq* OR jordan* OR libanaise OR Syria* OR turkey OR levant OR EMRO OR orient OR arabs OR arab OR arabia)) (61,121)

12 S6 AND S10 AND S11 (1,183)

-------------------------------------------

*Global Health Library Search Strategy*

1 (“health status” or “health transition” or “health level*” or “health need*” or “health demand*” or “health priorit*” or “health gap*” or “health service” or “health problem” or “health resource” or “health provider” or “health manpower” or “health training” or “ health education” or “health promotion” or “health development” or “health assessment” or doctor* or practitioner* or physician* or clinician* or nurse* or pharmacist* or dentist* or (disease (status or level or outbreak or transmission or prevalence or incidence or elimat* or eradicat*)) or (endemic (status or level or outbreak or transmission or prevalence or incidence or elimat* or eradicat*)) or (pandemic (status or level or outbreak or transmission or prevalence or incidence or elimat* or eradicat*)) or (pathogen (status or level or outbreak or transmission or prevalence or incidence or elimat* or eradicat*))) (4,917,418)

2 refugee* or emigrant* or immigrant* or transient* or nomad* or (displaced (individual or person or population)) or (relocated (individual or person or population)) or "vulnerable population" or "asylum seeker" or (war (victim* or survivor*)) or (conflict (victim or survivor*)) or exodus or "armed conflict victim*" or "armed conflict survivor*" or "disaster victim*" (437,978)

3 "middle east" OR "middle eastern" OR "eastern mediterranean" OR "east mediterranean" OR iraq or jordan* or lebanon or lebanese or syria or turkey or EMRO or orient or arabs or arab or arabia or levant (148,076)

4 #1 AND #2 AND #3 (3,657)
