# Supplementary material for: Assessment of the health needs of Syrian refugees in Lebanon and Syria’s neighboring countries
Source: Confl Health. 2019 Jun 27;13:31. doi: 10.1186/s13031-019-0211-3 (PMC6598365; doi:10.1186/s13031-019-0211-3)
Supplement: Supplementary file 3 — Appendix 3. Search strategies for grey literature (DOCX 85 kb) [file 13031_2019_211_MOESM3_ESM.docx]

**Appendix 3:** Search strategies for grey literature

*Search Strategy for Refworld*

Search One (90)

With all of the words: (health,doctor,practitioner,physician,clinician,nurse,pharmacist,dentist,disease,endemic,pandemic,pathogen)

Country of Origin: Syrian Arab Republic

Country of Asylum: Iraq

Search Two (143)

With all of the words: (health,doctor,practitioner,physician,clinician,nurse,pharmacist,dentist,disease,endemic,pandemic,pathogen)

Country of Origin: Syrian Arab Republic

Country of Asylum: Jordan

Search Three (163)

With all of the words: (health,doctor,practitioner,physician,clinician,nurse,pharmacist,dentist,disease,endemic,pandemic,pathogen)

Country of Origin: Syrian Arab Republic

Country of Asylum: Lebanon

Search Four (157)

With all of the words: (health,doctor,practitioner,physician,clinician,nurse,pharmacist,dentist,disease,endemic,pandemic,pathogen)

Country of Origin: Syrian Arab Republic

Country of Asylum: Turkey

----------------------------------------

*Search Strategy for WHO EMRO*

Searh (385)

(refugee) (lebanon OR syria OR iraq OR turkey OR jordan) site:www.emro.who.int

----------------------------------------

*Search Strategy for MSF*

Search One: Refugee Health, Syria (89 updates, 32 press)

Search Two: Refugee Health, Iraq (46 updates, 5 press)

Search Three: Refugee Health, Jordan (17 updates, 11 press)

Search Four: Refugee Health, Lebanon (30 updates, 3 press)

Search Five: Refugee Health, Turkey (17 updates, 3 press)

----------------------------------------

*Search Strategy for ICRC*

Refugee Health Needs

- Syria (351)
- Iraq (379)
- Jordan (76)
- Lebanon (205)
- Turkey (10)
