# Supplementary material for: Assessment of the health needs of Syrian refugees in Lebanon and Syria’s neighboring countries
Source: Confl Health. 2019 Jun 27;13:31. doi: 10.1186/s13031-019-0211-3 (PMC6598365; doi:10.1186/s13031-019-0211-3)
Supplement: Supplementary file 4 — Table S1. Excluded studies with reasons for exclusions from the database search. (DOCX 171 kb) [file 13031_2019_211_MOESM4_ESM.docx]

**Table S1. Excluded studies with reasons for exclusions from the database search**

| Study | Reason for Exclusion |
| --- | --- |
| Anonymous, 2012 (1) | Not the appropriate study design |
| Anonymous, 2014 (2) | Not the appropriate study design |
| Anonymous, 2016 (3) | Not the appropriate study design |
| Abbara et al., 2017 (4) | Not the appropriate study design |
| Abbara et al., 2016 (5) | Not the appropriate study design |
| Abbara et al., 2015 (6) | Not the appropriate study design |
| Abdella et al., 2017 (7) | Not the appropriate study design |
| Abo-Hilal et al., 2014 (8) | Not the appropriate outcome |
| Abou-Saleh et al., 2015 (9) | Not the appropriate study design |
| Acartuk et al., 2015 (10) | Not the appropriate outcome |
| Ahsan, 2013 (11) | Not the appropriate study design |
| Akbarzada et al., (12) | Not the appropriate study design |
| Akpinar et al., (13) | Full-text not found |
| Al Qutob, 2016 (14) | Not the appropriate study design |
| Al Qutob, 2016 (15) | Not the appropriate study design |
| Al-Ammouri et al., 2016 (16) | Not the appropriate population |
| Allesseri, 2013 (17) | Not the appropriate study design |
| Alsaba et al., 2016 (18) | Not the appropriate outcome |
| Al-Salem et al., 2016 (19) | Not the appropriate study design |
| Ammar et al., 2016 (20) | Not the appropriate outcome |
| Anonymous, 2015 (21) | Not the appropriate study design |
| Arafat et al., 2017 (22) | Not the appropriate population |
| Araj et al., 2016 (23) | Not the appropriate outcome |
| Aras et al., 2014 (24) | Not the appropriate population |
| Arie, 2015 (25) | Not the appropriate study design |
| Azer, 2016 (26) | Not the appropriate study design |
| Aziz et al., 2014 (27) | Not the appropriate outcome |
| Baarnhielm, 2016 (28) | Not the appropriate region |
| Barhoum et al., 2015 (29) | Not the appropriate region |
| Bashour, 2015 (30) | Not the appropriate study design |
| Beldjebel, 2014 (31) | Not the appropriate study design |
| Belen et al., 2016 (32) | Not the appropriate outcome |
| Ben Taleb et al., 2015 (33) | Not the appropriate study design |
| Berkley, 2017 (34) | Not the appropriate study design |
| Blanchet et al., 2016 (35) | Not the appropriate outcome |
| Budosan et al., 2016 (36) | Not the appropriate outcome |
| Buyuktiryaki et al., 2016 (37) | Not the appropriate study design |
| Buzzi et al., 2016 (38) | Not the appropriate study design |
| Cetorelli et al., 2017 (39) | Not the appropriate study design |
| Civaner et al., 2017 (40) | Not the appropriate outcome |
| Cochrane, 2015 (41) | Not the appropriate study design |
| Cookson et al., 2015 (42) | Not the appropriate outcome |
| Cousins, 2014 (43) | Not the appropriate study design |
| Cousins, 2015 (44) | Not the appropriate study design |
| Coutier, 2015 (45) | Not in English |
| Coutts et al., 2015 (46) | Not the appropriate study design |
| Coutts et al., 2013 (47) | Not the appropriate study design |
| Coutts et al., 2013 (48) | Not the appropriate study design |
| Davidson et al., 2016 (49) | Not the appropriate study design |
| DeJong, 2017 (50) | Not the appropriate study design |
| Demir et al., 2016 (51) | Not the appropriate population |
| Demirtas et al., 2015 (52) | Not the appropriate study design |
| Devi, 2012 (53) | Not the appropriate study design |
| Devi, 2016 (54) | Not the appropriate study design |
| Dewachi et al., 2014 (55) | Not the appropriate study design |
| Dikensoy et al., 2015 (56) | Not the appropriate study design |
| Doganay et al., 2016 (57) | Full-text not found |
| Dogru et al., 2017 (58) | Full-text not found |
| Doner et al., 2013 (59) | Not the appropriate study design |
| Doocy et al., 2015 (60) | Not the appropriate outcome |
| Doocy et al., 2017 (61) | Not the appropriate outcome |
| Doocy et al., 2015 (62) | Not the appropriate outcome |
| Dulger et al., 2015 (63) | Not the appropriate study design |
| Duramaz et al., 2017 (64) | Not the appropriate population |
| Eichner et al., 2013 (65) | Not the appropriate study design |
| Ekmkci, 2016 (66) | Not the appropriate outcome |
| El Chammay et al., 2014 (67) | Not the appropriate study design |
| El Chammay et al., 2016 (68) | Not the appropriate study design |
| El Kak, 2015 (69) | Full-text not found |
| El-Khani et al., 2016 (70) | Not the appropriate outcome |
| El-Khani et al., 2017 (71) | Not the appropriate outcome |
| El-Khatib et al., 2013 (72) | Not the appropriate study design |
| Eonomopoulou et al., 2017 (73) | Not the appropriate population |
| Feghali et al., 2017 (74) | Not the appropriate study design |
| Fouad et al., 2016 (75) | Not the appropriate study design |
| Fouad et al., 2017 (76) | Not the appropriate study design |
| Garfield, 2013 (77) | Not the appropriate study design |
| Gavlak, 2013 (78) | Not the appropriate study design |
| Gavlak, 2016 (79) | Not the appropriate study design |
| Ghandour, 2017 (80) | Full-text not found |
| Gulacti et al., 2017 (81) | Not the appropriate study design |
| Gulland, 2013 (82) | Not the appropriate outcome |
| Gunay et al., 2015 (83) | Not the appropriate study design |
| Habib, 2016 (84) | Not the appropriate study design |
| Habib et al., 2016 (85) | Not the appropriate population |
| Hampton, 2013 (86) | Not the appropriate study design |
| Hassan et al., 2016 (87) | Not the appropriate region |
| Heimer et al., 2017 (88) | Not the appropriate population |
| Heus et al., 2014 (89) | Not the appropriate population |
| His et al., 2013 (90) | Not the appropriate study design |
| Holmes, 2015 (91) | Not the appropriate study design |
| Hunter, 2016 (92) | Not the appropriate study design |
| Hurley, 2013 (93) | Not the appropriate study design |
| Inci et al., 2016 (94) | Not the appropriate study design |
| Inci et al., 2014 (95) | Not the appropriate population |
| Inci et al., 2016 (96) | Not in English |
| Ismail et al., 2016 (97) | Not the appropriate study design |
| Isreb et al., 2016 (98) | Not the appropriate study design |
| Jefee-Bahloul et al., 2016 (99) | Not the appropriate outcome |
| Jefee-Bahloul et al., 2015 (100) | Not the appropriate study design |
| Kaadan, 2014 (101) | Not the appropriate study design |
| Kara et al., 2017 (102) | Not the appropriate population |
| Kebudi et al., 2016 (103) | Not the appropriate study design |
| Koltas et al., 2014 (104) | Not the appropriate outcome |
| Kuey, 2016 (105) | Not the appropriate study design |
| Kutaish, 2015 (106) | Not the appropriate study design |
| Kutlu-Tonak, 2016 (107) | Not the appropriate outcome |
| Lam et al., 2017 (108) | Not the appropriate population |
| Langlois et al., 2016 (109) | Not the appropriate population |
| Leblebicioglu, 2016 (110) | Not the appropriate study design |
| Leblebiciolgu et al., 2015 (111) | Not the appropriate study design |
| Llosa et al., 2017 (112) | Not the appropriate population |
| Lough, 2015 (113) | Not the appropriate study design |
| Mahomed et al., 2016 (114) | Not the appropriate outcome |
| Mala et al., 2014 (115) | Not the appropriate study design |
| Maltezou et al., 2017 (116) | Not the appropriate region |
| Mbaeyi et al., 2017 (117) | Not the appropriate population |
| McKenzie et al., 2015 (118) | Not the appropriate population |
| Miller, 2016 (119) | Full-text not found |
| Moghaddam et al., 2017 (120) | Not the appropriate population |
| Morabia et al., 2015 (121) | Not the appropriate study design |
| Mulligan et al., 2015 (122) | Not the appropriate study design |
| Murshidi et al., 2013 (123) | Not the appropriate study design |
| Nasiroglu et al., 2016 (124) | Not the appropriate population |
| Nimri, 2016 (125) | Not the appropriate study design |
| Ouyang, 2013 (126) | Not the appropriate study design |
| Oymak et al., 2015 (127) | Not the appropriate study design |
| Ozaras et al., 2016 (128) | Not the appropriate study design |
| Ozdogan et al., 2016 (129) | Not the appropriate population |
| Ozdogan et al., 2014 (130) | Not the appropriate study design |
| Panter-Brick et al., 2017 (131) | Not the appropriate outcome |
| Parker, 2015 (132) | Not the appropriate study design |
| Petersen et al., 2013 (133) | Not the appropriate study design |
| Premkumer et al., 2016 (134) | Not the appropriate outcome |
| Qasaimeh et al., 2017 (135) | Not the appropriate population |
| Quosh, 2013 (136) | Not the appropriate population |
| Ramley et al., 2015 (137) | Not the appropriate study design |
| Refaat et al., 2013 (138) | Not the appropriate study design |
| Sahloul et al., 2016 (139) | Not the appropriate study design |
| Salman et al., 2014 (140) | Not in English |
| Samari, 2017 (141) | Not the appropriate study design |
| Sami et al., 2014 (142) | Not the appropriate study design |
| Sarikaya et al., 2016 (143) | Not the appropriate study design |
| Saroufim et al., 2015 (144) | Not the appropriate study design |
| Seita, 2016 (145) | Not the appropriate study design |
| Sekkarie et al., 2017 (146) | Not the appropriate region |
| Smeekes et al., 2017 (147) | Not the appropriate outcome |
| Song et al., 2016 (148) | Not the appropriate study design |
| Soydan et al., 2017 (149) | Not the appropriate population |
| Sozen Sahne et al., 2017 (150) | Not the appropriate population |
| Spiegel et al., 2014 (151) | Not the appropriate population |
| Stich, 2015 (152) | Not the appropriate study design |
| Sumpf et al., 2016 (153) | Not the appropriate outcome |
| Talhouk et al., 2017 (154) | Not the appropriate outcome |
| Talhouk et al., 2016 (155) | Not the appropriate outcome |
| Tas et al., 2017 (156) | Not the appropriate outcome |
| Tohme et al., 2016 (157) | Not the appropriate population |
| Tohme et al., 2016 (158) | Not the appropriate population |
| Turktan et al., 2017 (159) | Not the appropriate population |
| Tuzcu et al., 2015 (160) | Not the appropriate population |
| Uruc et al., 2014 (161) | Not the appropriate population |
| Usta et al., 2015 (162) | Not the appropriate study design |
| Vogel, 2013 (163) | Not the appropriate study design |
| Weinstein et al., 2016 (164) | Not the appropriate outcome |
| Wells et al., 2016 (165) | Not the appropriate study design |
| Wells et al., 2015 (166) | Not the appropriate outcome |
| Yazmine et al., 2016 (167) | Not the appropriate outcome |
| Yentur et al., 2016 (168) | Not in English |

1. A request to support the mental health needs of Syrian refugees. Intervention (15718883). 2012;10(3):234-6.

2. Syrian health crisis in Lebanon. The Lancet. 2014;383(9932):1862.

3. Reforming mental health in Lebanon amid refugee crises. Bull World Health Organ. 2016;94(8):564-5.

4. Abbara A, Al-Harbat N, Karah N, Abo-Yahya B, El-Amin W, Hatcher J, et al. Antimicrobial drug resistance among refugees from Syria, Jordan. Emerging Infectious Diseases. 2017;23(5):885-6.

5. Abbara A, Coutts A, Fouad FM, Ismail SA, Orcutt M. Mental Health among displaced Syrians: findings from the Syria Public Health Network. Journal of the Royal Society of Medicine. 2016;109(3):88-90.

6. Abbara A, Orcutt M, Gabbar O. Syria's lost generation of doctors. BMJ (Online). 2015;350.

7. Abdella Y, Hajjeh R, Smit Sibinga C. Availability and safety of blood transfusion during humanitarian emergencies. Vox Sanguinis. 2017;112:88-9.

8. Abo-Hilal M, Yousef OS. Beyond survival: A brief description of psychological services for: Syrian refugees. Peace and Conflict. 2014;20(3):334-6.

9. Abou-Saleh MT, Hughes P. Mental health of Syrian refugees: looking backwards and forwards. The lancet Psychiatry. 2015;2(10):870-1.

10. Acarturk C, Konuk E, Cetinkaya M, Senay I, Sijbrandij M, Cuijpers P, et al. EMDR for Syrian refugees with posttraumatic stress disorder symptoms: results of a pilot randomized controlled trial. European Journal of Psychotraumatology. 2015;6:27414.

11. Ahsan S. Providing medical relief in Syria's conflict. The Lancet. 2013;381(9866):523-4.

12. Akbarzada S, Mackey TK. The Syrian public health and humanitarian crisis: A ‘displacement’ in global governance? Global Public Health. 2017:1-17.

13. Akpinar F, Karahanoʇlu E, Kinay T, Coşkun B, Esin S, Mollamahmutoʇlu L. Guess who pays the bill? Of course the most innocent. Journal of the Turkish German Gynecology Association. 2016;17:S12.

14. Al Qutob MF. WHO and the refugee crisis in Jordan and beyond. The Lancet Global Health. 2016;4(5):e304.

15. Al Qutob MF. Refugees in Jordan. British dental journal. 2016;220(7):323.

16. Al-Ammouri I, Ayoub F. Heart Disease in Syrian Refugee Children: Experience at Jordan University Hospital. Annals of Global Health. 2016;82(2):300-6.

17. Alasseri N. Maxillofacial injuries experienced in support of Syrian freedom. International Journal of Oral and Maxillofacial Surgery. 2013;42(10):1218.

18. Alsaba K, Kapilashrami A. Understanding women’s experience of violence and the political economy of gender in conflict: the case of Syria. Reproductive Health Matters. 2016;24(47):5-17.

19. Al-Salem WS, Pigott DM, Subramaniam K, Haines LR, Kelly-Hope L, Molyneux DH, et al. Cutaneous Leishmaniasis and Conflict in Syria. Atlanta, Georgia: Centers for Disease Control & Prevention (CDC); 2016. p. 931-3.

20. Ammar W, Kdouh O, Hammoud R, Hamadeh R, Harb H, Ammar Z, et al. Health system resilience: Lebanon and the Syrian refugee crisis. Journal of Global Health. 2016;6(2):1-9.

21. Anonymous. Syrian doctors risk arrest and deportation for treating fellow refugees in Lebanon and Jordan.[Erratum for BMJ. 2015;350:h1552; PMID: 25801194]. BMJ. 2015;350:h1699.

22. Arafat S, Alsabek MB, Ahmad M, Hamo I, Munder E. Penetrating abdominal injuries during the Syrian war: Patterns and factors affecting mortality rates. Injury. 2017;48(5):1054-7.

23. Araj GF, Saade A, Itani LY, Avedissian AZ. Tuberculosis burden in Lebanon: Evolution and current status. Journal Medical Libanais. 2016;64(1):1-7.

24. Aras M, Altaş M, Yilmaz A, Serarslan Y, Yilmaz N, Yengil E, et al. Being a neighbor to Syria: A retrospective analysis of patients brought to our clinic for cranial gunshot wounds in the Syrian civil war. Clinical Neurology and Neurosurgery. 2014;125:222-8.

25. Arie S. Syrian doctors risk deportation for treating fellow refugees in Lebanon and Jordan. BMJ: British Medical Journal. 2015;350(8001):h1552-h.

26. Azer SA. Supporting Syrian Refugees: The Need for a Multidisciplinary Action Plan. American journal of public health. 2016;106(5):e18-e9.

27. Aziz IA, Hutchinson CV, Maltby J. Quality of life of Syrian refugees living in camps in the Kurdistan region of Iraq. PeerJ. 2014;2014(1).

28. Bäärnhielm S. Refugees' mental health--a call for a public health approach with focus on resilience and cultural sensitivity. European Journal of Public Health. 2016;26(3):375-6.

29. Barhoum M, Tobias S, Elron M, Sharon A, Heija T, Soustiel JF. Syria civil war: Outcomes of humanitarian neurosurgical care provided to Syrian wounded refugees in Israel. Brain Injury. 2015;29(11):1370-5.

30. Bashour H. Let's Not Forget the Health of the Syrians Within Their Own Country. American Journal of Public Health. 2015;105(12):2407-8.

31. Beldjebel I. Infectious diseases in refugees coming from Syria and Iraq to Lebanon. International Journal of Infectious Diseases. 2014;21:26.

32. Belen BF, Polat M, Özsevik SN, Soylu E. Frequency of neutropenia among Turkish and Syrian pediatric thalassemia patients under deferiprone monotherapy. Pediatric Hematology and Oncology. 2016;33(1):51-8.

33. Ben Taleb Z, Bahelah R, Fouad FM, Coutts A, Wilcox M, Maziak W. Syria: health in a country undergoing tragic transition. International journal of public health. 2015;60:S63-S72.

34. Berkley S. Syria, slums, and health security. Science. 2017;356(6336):353.

35. Blanchet K, Fouad FM, Pherali T. Syrian refugees in Lebanon: the search for universal health coverage. Confl Health. 2016;10:12-.

36. Budosan B, Aziz S, Benner MT, Abras B. Perceived needs and daily stressors in an urban refugee setting: Humanitarian Emergency Settings Perceived Needs Scale survey of Syrian refugees in Kilis, Turkey. Intervention (15718883). 2016;14(3):293-304.

37. Büyüktiryaki M, Canpolat FE, Alyamaç Dizdar E, Okur N, Kadioäÿlu Şimşek G. Neonatal outcomes of Syrian refugees delivered in a tertiary hospital in Ankara, Turkey. Conflict and Health. 2015;9(1).

38. Buzzi F, Rossi A, Serra I, Corrado R, Soliman C, Chiodi Daelli F, et al. Nursing care of children with thalassemia during the Syrian Crisis an Italian experience in the autonomous region of Kurdistan-Iraq. Bone Marrow Transplantation. 2016;51:S514.

39. Cetorelli V, Sasson I, Shabila N, Burnham G. Mortality and kidnapping estimates for the Yazidi population in the area of Mount Sinjar, Iraq, in August 2014: A retrospective household survey. PLoS Medicine. 2017;14(5):1-15.

40. Civaner MM, Vatansever K, Pala K. Ethical problems in an era where disasters have become a part of daily life: A qualitative study of healthcare workers in Turkey. PLoS ONE. 2017;12(3).

41. Cochrane P. SUPPORT IN A TIME OF PERIL. Nursing Standard. 2015;29(48):64-5.

42. Cookson ST, Abaza H, Clarke KR, Burton A, Sabrah NA, Rumman KA, et al. &quot;Impact of and response to increased tuberculosis prevalence among Syrian refugees compared with Jordanian tuberculosis prevalence: case study of a tuberculosis public health strategy&quot. Confl Health. 2015;9:18-.

43. Cousins S. Experts sound alarm as Syrian crisis fuels spread of tuberculosis. BMJ (Online). 2014;349.

44. Cousins S. Syrian crisis: health experts say more can be done. Lancet. 2015;385 North American Edition(9972):931-4.

45. Coutier D. Syrian crisis: Looks on the psychological support of the refugees. Annales Medico-Psychologiques. 2015;173(10):855-8.

46. Coutts A, Fouad FM, Abbara A, Sibai AM, Sahloul Z, Blanchet K. Responding to the Syrian health crisis: The need for data and research. The Lancet Respiratory Medicine. 2015;3(3):e8-e9.

47. Coutts A, Fouad FM, Batniji R. Assessing the Syrian health crisis: The case of Lebanon. The Lancet. 2013;381(9875):e9.

48. Coutts A, McKee M, Stuckler D. The emerging Syrian health crisis. Lancet. 2013;381 North American Edition(9865):e6-7.

49. Davidson PM, Mbaka-Mouyeme F. The refugee crisis: We cannot ignore this for much longer. Health Care for Women International. 2016;37(9):945-.

50. DeJong J. Challenges to understanding the reproductive health needs of women forcibly displaced by the Syrian conflict. J Fam Plann Reprod Health Care. 2017;43(2):103-4.

51. Demir D, Abanoz M, Tulay CM, Aydın MS, Kasapoğlu BÖ, Merdanoğlu M, et al. Outcomes of coronary artery bypass surgery in syrian refugees. International Journal of Clinical and Experimental Medicine. 2016;9(7):13195-9.

52. Demirtas U, Ozden A. Syrian refugees: health services support and hospitality in Turkey. Public Health (Elsevier). 2015;129(11):1549-50.

53. Devi S. Syria's refugees face a bleak winter. Lancet. 2012;380 North American Edition(9851):1373-4.

54. Devi S. Syria's health crisis: 5 years on. Lancet. 2016;387 North American Edition(10023):1042-3.

55. Dewachi O, Skelton M, Nguyen VK, Fouad FM, Sitta GA, Maasri Z, et al. Changing therapeutic geographies of the Iraqi and Syrian wars. The Lancet. 2014;383(9915):449-57.

56. Dikensoy O, Bayram H, Uyar M, Light RW. Being a pulmonary physician nearby a war zone: Syrian patients admitted to a tertiary hospital in gaziantep province. American Journal of Respiratory and Critical Care Medicine. 2015;191.

57. Doganay M, Demiraslan H. Refugees of the Syrian Civil War: Impact on Reemerging Infections, Health Services, and Biosecurity in Turkey. Health security. 2016;14(4):220-5.

58. Doğru S, Döner P. Frequency and outcomes of new patients with pulmonary tuberculosis in Hatay province after Syrian civil war. Indian Journal of Tuberculosis. 2017;64(2):83-8.

59. Döner P, Özkara A, Kahveci R. Syrian refugees in Turkey: Numbers and emotions. The Lancet. 2013;382(9894):764.

60. Doocy S, Delbiso TD, Team TIS, Guha-Sapir D. The humanitarian situation in Syria: A snapshot in the third year of the crisis. PLoS Currents. 2015;7(DISASTERS).

61. Doocy S, Lyles E. Humanitarian Needs Among Displaced and Female-Headed Households in Government-Controlled Areas of Syria. American journal of public health. 2017;107(6):950-9.

62. Doocy S, Lyles E, Delbiso TD, Robinson CW. Internal displacement and the Syrian crisis: an analysis of trends from 2011-2014. Confl Health. 2015;9:33-.

63. Dülger AC, Çolak B, Saʇcan M, Türkdoʇan K, Kotan C. Hepatitis B prevalence among Syrian immigrants in Turkey. Hepatology International. 2015;9(1):S211-S2.

64. Duramaz A, Bilgili MG, Bayram B, Ziroglu N, Bayrak A, Avkan MC. Orthopedic trauma surgery and hospital cost analysis in refugees; the effect of the Syrian civil War. Int Orthop. 2017;41(5):877-84.

65. Eichner M, Brockmann SO. Polio emergence in Syria and Israel endangers Europe. Lancet. 2013;382 North American Edition(9907):1777-.

66. Ekmekci PE. Syrian Refugees, Health and Migration Legislation in Turkey. 2016.

67. El Chammay R, Ammar W. Syrian crisis and mental health system reform in Lebanon. Lancet. 2014;384 North American Edition(9942):494-.

68. El Chammay R, Karam E, Ammar W. Mental health reform in Lebanon and the Syrian crisis. The Lancet Psychiatry. 2016;3(3):202-3.

69. El Kak F. Challenges in reproductive health in refugee camps in limited resources settings-the experiences from Lebanon. International Journal of Gynecology and Obstetrics. 2015;131:E5.

70. El-Khani A, Cartwright K, Redmond A, Calam R. Daily bread: a novel vehicle for dissemination and evaluation of psychological first aid for families exposed to armed conflict in Syria. Glob Ment Health (Camb). 2016;3:e15-e.

71. El-Khani A, Ulph F, Peters S, Calam R. Syria: coping mechanisms utilised by displaced refugee parents caring for their children in pre-resettlement contexts. Intervention (15718883). 2017;15(1):34-50.

72. El-Khatib Z, Scales D, Vearey J, Forsberg BC. Syrian refugees, between rocky crisis in Syria and hard inaccessibility to healthcare services in Lebanon and Jordan. Conflict & Health [Electronic Resource]. 2013;7(1):18.

73. Eonomopoulou A, Pavli A, Stasinopoulou P, Giannopoulos LA, Tsiodras S. Migrant screening: Lessons learned from the migrant holding level at the Greek–Turkish borders. Journal of Infection and Public Health. 2017;10(2):177-84.

74. Feghali R, Zaugg C. Lebanese red cross blood transfusion services: Scaling up its activities to satisfy blood needs of syrian refugees and host population in lebanon. Vox Sanguinis. 2017;112:77.

75. Fouad FM, Alameddine M, Coutts A. Human resources in protracted crises: Syrian medical workers. The Lancet. 2016;387(10028):1613.

76. Fouad FM, Sparrow A, Tarakji A, Alameddine M, El-Jardali F, Coutts AP, et al. Health workers and the weaponisation of health care in Syria: A preliminary inquiry for The Lancet-American University of Beirut Commission on Syria. The Lancet. 2017.

77. Garfield R. Health professionals in Syria. Lancet. 2013;382 North American Edition(9888):205-6.

78. Gavlak D. Syrians flee violence and disrupted health services to Jordan. Bulletin of the World Health Organization. 2013;91(6):394-5.

79. Gavlak D. Healing invisible wounds of the Syrian conflict. Bulletin of the World Health Organization. 2016;94(1):6-7.

80. Ghandour LA, El Hayek G, Yunis K. Perinatal and neonatal mortality in conflict settings: Evidence-based interventions to reduce adverse birth outcomes. Neonatal and Perinatal Mortality: Global Challenges, Risk Factors and Interventions2017. p. 171-86.

81. Gulacti U, Lok U, Polat H. Emergency department visits of Syrian refugees and the cost of their healthcare. Pathogens and Global Health. 2017:1-6.

82. Gulland A. Syrian refugees in Lebanon find it hard to access healthcare, says charity. BMJ (Clinical research ed). 2013;346.

83. Gunay E, Acar K. Syrian refugees at a distant emergency department in Turkey. Academic Emergency Medicine. 2015;22(5):S209-S10.

84. Habib RR. Syrian labour in Lebanon: Review of current trends in the literature. Occupational and Environmental Medicine. 2016;73:A186.

85. Habib RR, Mikati D, Hojeij S, El Asmar K, Chaaya M, Zurayk R. Associations between poor living conditions and multi-morbidity among Syrian migrant agricultural workers in Lebanon. European Journal of Public Health. 2016;26(6):1039-44.

86. Hampton T. Health care under attack in Syrian conflict. JAMA. 2013;310(5):465-6.

87. Hassan G, Ventevogel P, Jefee-Bahloul H, Barkil-Oteo A, Kirmayer LJ. Mental health and psychosocial wellbeing of Syrians affected by armed conflict. Epidemiology and Psychiatric Sciences. 2016;25(2):129-41.

88. Heimer R, Barbour R, Khouri D, Crawford FW, Shebl F, Aaraj E, et al. HIV Risk, Prevalence, and Access to Care Among Men Who Have Sex with Men in Lebanon. 2017.

89. Heus K, Sartawi T. The realization of the right to health for refugees in Jordan. The Right to Health: A Multi-Country Study of Law, Policy and Practice2014. p. 193-229.

90. His E, Murshidi MM, Hijjawi MQ, Jeriesat S, Eltom A. Syrian refugees and Jordan's health sector. Lancet. 2013;382(9888):206-7.

91. Holmes D. Chronic disease care crisis for Lebanon's Syrian refugees. The Lancet Diabetes and Endocrinology. 2015;3(2):102.

92. Hunter P. The refugee crisis challenges national health care systems: Countries accepting large numbers of refugees are struggling to meet their health care needs, which range from infectious to chronic diseases to mental illnesses. EMBO Reports. 2016;17(4):492-5.

93. Hurley R. Who cares for the nine million displaced people of Syria? BMJ (Online). 2013;347.

94. Inci A, Yildirim D, Ülker V, Akbayir Ö, Numanoǧlu C. The investigation of HBsAg, anti HCV and anti HIV frequency in turkish and syrian refugees who applied to gynecology-oncology outpatient clinic. International Journal of Gynecological Cancer. 2016;26(2):39-40.

95. Inci M, Karakuş A, Rifaioglu MM, Yengil E, Atçi N, Akin Ö, et al. A practice report of bladder injuries due to gunshot wounds in Syrian refugees. Ulusal Travma ve Acil Cerrahi Dergisi. 2014;20(5):371-5.

96. Inci R, Öztürk P, Mülayim MK, Karakuzu A, Kelekçi KH, Inci MF, et al. Dermatological face of Syrian civil war. Turkderm Deri Hastaliklari ve Frengi Arsivi. 2016;50(4):145-9.

97. Ismail SA, Abbara A, Collin SM, Orcutt M, Coutts AP, Maziak W, et al. Communicable disease surveillance and control in the context of conflict and mass displacement in Syria. Int J Infect Dis. 2016;47:15-22.

98. Isreb M, Al Kukhun H, Al-Adwan SAS, Kass-Hout TA, Murad L, Rifai AO, et al. Psychosocial impact of war on syrian refugees with ESRD. American Journal of Kidney Diseases. 2016;67(5):A56.

99. Jefee-Bahloul H, Barkil-Oteo A, Shukair N, Alraas W, Mahasneh W. Using a Store-and-Forward System to Provide Global Telemental Health Supervision and Training: A Case from Syria. Academic psychiatry : the journal of the American Association of Directors of Psychiatric Residency Training and the Association for Academic Psychiatry. 2016;40(4):707-9.

100. Jefee-Bahloul H, Cohen S, Bitar A, Kairuz C, Terepka A, Kanawati Y, et al. Psychosocial functioning of Syrian child refugees in Jordan and Turkey. European Child and Adolescent Psychiatry. 2015;24(1):S9.

101. Kaadan A. The effect of civil war on cutaneous leishmaniasis (“aleppo button”) in aleppo city, syria. American Journal of Tropical Medicine and Hygiene. 2014;91(5):330.

102. Kara MA, Kılıç BD, Çöl N, Özçelik AA, Büyükçelik M, Balat A. Kidney disease profile of Syrian refugee children. Iranian Journal of Kidney Diseases. 2017;11(2):109-14.

103. Kebudi R, Bayram I, Yagci Kupeli B, Kupeli S, Sezgin G, Pekpak E, et al. Cancer in refugee children in Turkey. Pediatric Blood and Cancer. 2016;63:S41.

104. Koltas IS, Eroglu F, Alabaz D, Uzun S. The emergence of Leishmania major and Leishmania donovani in southern Turkey. Transactions of the Royal Society of Tropical Medicine and Hygiene. 2014;108(3):154-8.

105. Küey L. A new humanitarian emergency: Refugees and mental health in Turkey. European Psychiatry. 2016;33:S9.

106. Kutaish H. Health of Syrian refugees role of brucellosis in febrile diseases among the refugee population. Tropical Medicine and International Health. 2015;20:396.

107. Kutlu-Tonak Z. Endless Escape: From Syria to Turkey, Then to Europe. Studies in Ethnicity and Nationalism. 2016;16(1):121-34.

108. Lam E, Al-Tamimi W, Russell SP, Butt MO-UI, Blanton C, Musani AS, et al. Oral Cholera Vaccine Coverage during an Outbreak and Humanitarian Crisis, Iraq, 2015. Emerg Infect Dis. 2017;23(1):38-45.

109. Langlois EV, Haines A, Tomson G, Ghaffar A. Refugees: Towards better access to health-care services. The Lancet. 2016;387(10016):319-21.

110. Leblebicioglu H. Managing health and infections in refugees: Turkey's experience. International Journal of Infectious Diseases. 2016;45:56.

111. Leblebicioglu H, Ozaras R. Syrian refugees and infectious disease challenges. Travel Medicine and Infectious Disease. 2015;13(6):443-4.

112. Llosa AE, Van Ommeren M, Kolappa K, Ghantous Z, Souza R, Bastin P, et al. A two-phase approach for the identification of refugees with priority need for mental health care in Lebanon: A validation study. BMC Psychiatry. 2017;17(1).

113. Lough S. Health agencies overwhelmed by Syrian crisis. CMAJ: Canadian Medical Association Journal. 2015;187(16):1196-.

114. Mahomed Z, Motara F, Bham A. Humanitarian Medical Response to the Syrian Arab Republic (April 7, 2013 to April 23, 2013). Prehospital and disaster medicine. 2016;31(1):114-6.

115. Mala P, Ghada M, Wasan A, Noor AA, Alhamadani AS, Gmach S, et al. Establishment of EWARN system for the Syrian crisis: Experiences and challenges. International Journal of Infectious Diseases. 2014;21:280.

116. Maltezou HC, Theodoridou M, Daikos GL. Antimicrobial resistance and the current refugee crisis. Journal of Global Antimicrobial Resistance. 2017;10:75-9.

117. Mbaeyi C, Ryan MJ, Smith P, Mahamud A, Farag N, Haithami S, et al. Response to a Large Polio Outbreak in a Setting of Conflict - Middle East, 2013-2015. MMWR: Morbidity & Mortality Weekly Report. 2017;66(8):227-31.

118. McKenzie E, Spiegel P, Khalifa A, Mateen F. Neuropsychiatric disorders among Syrian and Iraqi refugees in Jordan, 2012-2013: A retrospective, cohort study. Neurology. 2015;84.

119. Miller SD. Political and humanitarian responses to syrian displacement2016. 1-146 p.

120. Moghaddam HT, Sayedi SJ, Moghadam ZE, Bahreini A, Abbasi MA, Saeidi M. Refugees in the Eastern Mediterranean Region: Needs, problems and challenges. International Journal of Pediatrics. 2017;5(3):4625-39.

121. Morabia A, Benjamin GC. The Refugee Crisis in the Middle East and Public Health. American Journal of Public Health. 2015;105(12):2405-6.

122. Mulligan CJ, Clukay C, Quinlan J, Dajani R, Hamadmad D, Abudayyeh G, et al. Genetics of risk and resilience in Syrian refugee youth. American Journal of Physical Anthropology. 2017;162:294-5.

123. Murshidi MM, Hijjawi MQB, Jeriesat S, Eltom A. Syrian refugees and Jordan&#039;s health sector. Lancet. 2013;382(9888):206-7.

124. Nasıroğlu S, Çeri V. Posttraumatic stress and depression in Yazidi refugees. Neuropsychiatric Disease and Treatment. 2016;12:2941-8.

125. Nimri O. Influx of foreign refugees to Jordan and its overall burden on cancer care. Cancer Care in Countries and Societies in Transition: Individualized Care in Focus2016. p. 99-104.

126. Ouyang H. Syrian refugees and sexual violence. The Lancet. 2013;381(9884):2165-6.

127. Oymak Y, Ince D, Demirag B, Ali B, Yaman Y, Ozek G, et al. Cancer in Syrian refugee children. Pediatric Blood and Cancer. 2015;62:S294-S5.

128. Ozaras R, Leblebicioglu H, Sunbul M, Tabak F, Balkan II, Yemisen M, et al. The Syrian conflict and infectious diseases. Expert Review of Anti-Infective Therapy. 2016;14(6):547-55.

129. Ozdogan HK, Karateke F, Ozdogan M, Cetinalp S, Ozyazici S, Gezercan Y, et al. The Syrian civil war: The experience of the Surgical Intensive Care Units. Pak J Med Sci. 2016;32(3):529-33.

130. Ozdogan HK, Karateke F, Ozdogan M, Satar S. Syrian refugees in Turkey: Effects on intensive care. The Lancet. 2014;384(9952):1427-8.

131. Panter-Brick C, Hadfield K, Dajani R, Eggerman M, Ager A, Ungar M. Resilience in Context: A Brief and Culturally Grounded Measure for Syrian Refugee and Jordanian Host-Community Adolescents. 2017.

132. Parker S. Hidden crisis: Violence against Syrian female refugees. The Lancet. 2015;385(9985):2341-2.

133. Petersen E, Baekeland S, Memish ZA, Leblebicioglu H. Infectious disease risk from the Syrian conflict. International Journal of Infectious Diseases. 2013;17(9):e666-e7.

134. Premkumar A, Raad K, Haidar MH. Rethinking the social history in the era of biolegitimacy: global health and medical education in the care of Palestinian and Syrian refugees in Beirut, Lebanon. Anthropology & Medicine. 2016;23(1):14-29.

135. Qasaimeh GR, Shotar AM, Alkhail SJA, Qasaimeh MG. The pattern of the Syrian refugee’s injuries managed in King Abdullah University Hospital (Jordan). European Journal of Trauma and Emergency Surgery. 2017:1-8.

136. Quosh C. Mental health, forced displacement and recovery: integrated mental health and psychosocial support for urban refugees in Syria. Intervention (15718883). 2013;11(3):295-320.

137. Ramly EP, Rahbani DDR, Khalifeh J, Hoballah JJ, Kaafarani HMA. Systematic evaluation of national surgical capacity in Lebanon in times of crisis and refugees. Journal of the American College of Surgeons. 2015;221(4):S89.

138. Refaat MM, Mohanna K. Syrian refugees in Lebanon: Facts and solutions. The Lancet. 2013;382(9894):763-4.

139. Sahloul MZ, Monla-Hassan J, Sankari A, Kherallah M, Atassi B, Badr S, et al. War is the Enemy of Health. Pulmonary, Critical Care, and Sleep Medicine in War-Torn Syria. Annals of the American Thoracic Society. 2016;13(2):147-55.

140. Salman IS, Vural A, Unver A, Sacar S. [Cutaneous leishmaniasis cases in Nizip, Turkey after the Syrian civil war]. Mikrobiyoloji Bulteni. 2014;48(1):106-13.

141. Samari G. Syrian Refugee Women's Health in Lebanon, Turkey, and Jordan and Recommendations for Improved Practice. World Medical and Health Policy. 2017;9(2):255-74.

142. Sami S, Williams HA, Krause S, Onyango MA, Burton A, Tomczyk B. Responding to the Syrian crisis: the needs of women and girls. Lancet. 2014;383 North American Edition(9923):1179-81.

143. Sarikaya E, Durutuna S, Kapisiz SG, Erkaya S, Sarikaya DA. Five years data about refugee services of Turkey's biggest government woman hospital. Journal of the Turkish German Gynecology Association. 2016;17:S18-S9.

144. Saroufim M, Charafeddine K, Khalifeh I. A new face of cutaneous leishmaniasis in a time of war. Laboratory Investigation. 2014;94:393A.

145. Seita A. Complex emergencies in the Eastern Mediterranean Region: Impact on tuberculosis control. International Journal of Mycobacteriology. 2016;5:S12.

146. Sekkarie MA, Abdel-Rahman EM. Cultural Challenges in the Care of Refugees with End-Stage Renal Disease: What Western Nephrologists Should Know. Nephron. 2017.

147. Smeekes A, Verkuyten M, Çelebi E, Acartürk C, Onkun S. Social identity continuity and mental health among Syrian refugees in Turkey. 2017.

148. Song SJ. Evidence base of resilience in war-affected youth: Syrian refugee adolescents. Journal of the American Academy of Child and Adolescent Psychiatry. 2016;55(10):S6.

149. Soydan L, Demir AA, Tunaci A. Frequency of abnormal pulmonary computed tomography findings in asylum seeking refugees in Turkey. International Health. 2017;9(2):118-23.

150. Sözen Şahne B, Arslan M, Şar S. Health and pharmacy services for refugees in Turkey. Fabad Journal of Pharmaceutical Sciences. 2015;40(1):27-31.

151. Spiegel P, Khalifa A, Mateen FJ. Cancer in refugees in Jordan and Syria between 2009 and 2012: Challenges and the way forward in humanitarian emergencies. The Lancet Oncology. 2014;15(7):e290-e7.

152. Stich A. Coming in to the cold – Access to health care is urgently needed for Syrian refugees. Travel Medicine & Infectious Disease. 2015;13(6):445-6.

153. Sumpf D, Isaila V, Najjar K, editors. The impact of the Syria crisis on Lebanon. Springer Proceedings in Mathematics and Statistics; 2016.

154. Talhouk R, Bartindale T, Montague K, Mesmar S, Akik C, Ghassani A, et al., editors. Implications of synchronous IVR radio on Syrian refugee health and community dynamics. ACM International Conference Proceeding Series; 2017.

155. Talhouk R, Mesmar S, Thieme A, Balaam M, Olivier P, Akik C, et al., editors. Syrian refugees and digital health in Lebanon: Opportunities for improving antenatal health. Conference on Human Factors in Computing Systems - Proceedings; 2016.

156. Tas B, Kulacaoglu F, Altuntas M. Effects of sociodemographic sexual and clinical factors and disease awareness on psychosexual dysfunction of refugee patients with anogenital warts in Turkey: A cross-sectional study. Biomedical Research (India). 2017;28(12):5601-8.

157. Tohme J, Egan J, Friedman M, Stall R. Psycho-social Correlates of Condom Use and HIV Testing among MSM Refugees in Beirut, Lebanon. AIDS & Behavior. 2016;20:417-25.

158. Tohme J, Egan JE, Stall R, Wagner G, Mokhbat J. HIV Prevalence and Demographic Determinants of Unprotected Anal Sex and HIV Testing among Male Refugees Who have Sex with Men in Beirut, Lebanon. AIDS Behav. 2016;20(Suppl 3):408-16.

159. Turktan M, Ak O, Erdem H, Ozcengiz D, Hargreaves S, Kaya S, et al. Community acquired infections among refugees leading to Intensive Care Unit admissions in Turkey. International Journal of Infectious Diseases. 2017;58:111-4.

160. Tuzcu A, Bahar Z. Barriers and Facilitators to Breast Cancer Screening Among Migrant Women Within Turkey. Journal of Transcultural Nursing. 2015;26(1):47-56.

161. Uruc V, Ozden R, Duman IG, Dogramaci Y, Yengil E, Karapinar S, et al. Major musculoskeletal injuries and applied treatments in the current conflicts in Syria. Acta Medica Mediterranea. 2014;30(3):637-44.

162. Usta J, Masterson AR. Women and health in refugee settings: The case of displaced syrian women in Lebanon. Gender-Based Violence: Perspective from Africa, the Middle East, and India2015. p. 119-43.

163. Vogel L. WHO releases guidelines for treating chemical warfare victims after possible Syria attacks. CMAJ : Canadian Medical Association journal = journal de l'Association medicale canadienne. 2013;185(14):E665.

164. Weinstein N, Khabbaz F, Legate N. Enhancing Need Satisfaction to Reduce Psychological Distress in Syrian Refugees. Journal of Consulting and Clinical Psychology. 2016;84(7):645-50.

165. Wells R, Steel Z, Abo-Hilal M, Hassan AH, Lawsin C. Psychosocial concerns reported by Syrian refugees living in Jordan: Systematic review of unpublished needs assessments. British Journal of Psychiatry. 2016;209(2):99-106.

166. Wells R, Wells D, Lawsin C. Understanding psychological responses to trauma among refugees: The importance of measurement validity in cross-cultural settings. Journal and Proceedings of the Royal Society of New South Wales. 2015;148(455-456):60-9.

167. Yasmine R, Moughalian C. Systemic violence against Syrian refugee women and the myth of effective intrapersonal interventions. Reproductive Health Matters. 2016;24(47):27-35.

168. Yentur Doni N, Aksoy M, Simsek Z, Gurses G, Hilali NG, Yildiz Zeyrek F, et al. [Investigation of the prevalence of Trichomonas vaginalis among female Syrian refugees with the complaints of vaginitis aged between 15-49 years]. Mikrobiyoloji Bulteni. 2016;50(4):590-7.
