# Supplementary material for: Assessment of the health needs of Syrian refugees in Lebanon and Syria’s neighboring countries
Source: Confl Health. 2019 Jun 27;13:31. doi: 10.1186/s13031-019-0211-3 (PMC6598365; doi:10.1186/s13031-019-0211-3)
Supplement: Supplementary file 5 — Table S2. Summary of studies included for analysis from the database search. (DOCX 101 kb) [file 13031_2019_211_MOESM5_ESM.docx]

**Table S2. Summary of studies included for analysis from the database search**

| First Author | Design | Country | Research Questions Addressed* | Findings |
| --- | --- | --- | --- | --- |
| Abo-Hilal et al. (1) | Qualitative Case Study | Jordan | *2, 3, 4* | - Lack of mental health services overall. - Need for training relating to basic skills for referring psychological problems. |
| Acarturk et al. (2) | Quantitative Cross-Sectional Study | Turkey | *1* | - High prevalence of PTSD (83%) and depression (37%). |
| Akoury-Dirani et al. (3) | Quantitative Case study | Lebanon | *3* | - Need for training for delivery appropriate psychological first aid. |
| Al Qadire et al. (4) | Quantitative Cross-Sectional Study | Jordan | *2* | - Gap in seeking medical care for cancer symptoms; did not have insurance (83%), worried what might be found (77%), embarrassed (61%), scared (56%), could not get an appointment (52%) |
| Alawieh et al. (5) | Quantitative Case Study | Lebanon | *1* | - 1033 new cases of Leishmaniosis; almost all among Syrian refugees (97%). |
| Al-Fahoum et al. (6) | Quantitative Cross-Sectional Study | Jordan | *1, 2* | - Hypertension (30%) and asthma (30%) were the main health problems for women; hypertension (41%) for men. - Insufficient health care services, concerns regarding access to nutrition. - Perception of insufficient health care services (75%), access to nutrition (64%). |
| Alnuaimi et al. (7) | Quantitative Case Study | Jordan | *1* | - High rates of anemia (51%) and caesarean sections (37%) among Syrian women. |
| Alpak et al. (8) | Quantitative Cross-Sectional Study | Turkey | *1* | - High prevalence of PTSD (34%). |
| Al-Smadi et al. (9) | Quantitative Cross-Sectional Study | Jordan | *1* | - Above average prevalence of PTSD (56%). |
| Ay et al. (10) | Quantitative Cross-Sectional Study | Jordan | *2* | - Service needs assessment: Perceived insufficiencies for PHC for acute diseases (57%), dental (40%), vaccinations (40%), chronic diseases (37%), OBGYN (33%), emergency care (25%). |
| Basheti et al. (11) | Quantitative Cross-Sectional Study | Jordan | *1, 2* | - High prevalence of suffering from some form of psychological distress (56%). - Reported high need for psychological support (46%). |
| Benage et al. (12) | Quantitative Cross-Sectional Study | Lebanon | *1, 2* | - High need for antenatal care, low prevalence of adequate diet of vitamins for pregnant women (41%). - Low amount had received all 3 necessary antenatal visits (31%). |
| Bilukha et al. (13) | Quantitative Cross-Sectional Study | Jordan | *1* | - Acute malnutrition relatively low (1%), global malnutrition average (9 – 17%). |
| Bouchghoul et al. (14) | Quantitative Case Study | Jordan | *1, 2* | - Low maternal (4%) and neonatal morbidity (5%). - Most women with antenatal complications were successfully referred to a hospital (88%). |
| Bucak et al. (15) | Quantitative Cross-Sectional Study | Turkey | *1* | - High prevalence of anemia (50%), chronic malnutrition (19%). |
| Cartwright et al. (16) | Quantitative Cross-Sectional Study | Turkey | *1* | - High prevalence of emotional distress in children (49% anxious, 62% fearful). |
| Cetorelli et al. (17) | Quantitative Cross-Sectional Study | Iraq | *1, 2* | - Hypertension prevalence rose from 4.4% among those aged 30–44 to 23.9% among those aged 45–59, and reached 32.1% among those aged 60 and above.   - Prevalence of musculoskeletal conditions was 4.2% among those aged 30–44, rising to 12.3% among those aged 45–59 and to 22.4% among those aged 60 and over - Sufficient services for NCDs (95% had seen a healthcare provider for an NCD in the past 3 months). |
| Chemali et al. (18) | Quantitative Case Study | Lebanon | *3* | - Need for self-help skills to ensure the mental well-being of field workers. |
| Cherri et al. (19) | Qualitative Case Study | Lebanon | *2* | - Main barriers to accessing contraceptives was cost, being unaware of services, and their husbands’ refusal. |
| Chung et al. (20) | Quantitative Cross-Sectional Study | Turkey | *1* | - High prevalence of PSD (52%). |
| Collins et al. (21) | Mixed Methods Cross-Sectional Study | Jordan | *1, 3* | - High prevalence of risk scores for CVDs (23%); high prevalence of history of CVDs (21%). - Need for training to properly identify and report CVD risk scores. |
| Demirci et al. (22) | Quantitative Cross-Sectional Study | Turkey | *1* | - Caesarean rates were high (32%), still lower than Turkish citizens (43%). |
| Doocy et al. (23) | Quantitative Cross-Sectional Study | Jordan | *1, 2* | - Health needs assessment; highest was communicable diseases (22%), NCDs (21%), injuries (10%). - Main barrier to not seeking care was costs (65%). |
| Doocy et al. (24) | Quantitative Cross-Sectional Study | Lebanon | *1, 2* | - High prevalence of hypertension (21%), CVDs (11%), diabetes (10%). - Syrians receive NCD care access at the same frequency as Lebanese host community members. |
| Doocy et al. (25) | Quantitative Cross-Sectional Study | Jordan | *1, 2* | - High prevalence of reported households with NCDs (50%); hypertension (11%), arthritis (7%), diabetes (6%). - High care seeking behavior for NCD treatment (85%); main barrier was cost. |
| Doocy et al. (26) | Cohort Study | Lebanon | *2* | - Perceived low interest in patient on provider side; did not ask about medication complications (50%), or general questions (60%). |
| Duzkoylu et al. (27) | Quantitative Case-Control Study | Turkey | *1* | - Higher emergency admissions of Syrian refugees; sprain of extremities (37%), skin tear (21%), and head injuries (19%). |
| Elamein et al. (28) | Quantitative Case Study | Syria | *4* | - Targeted attacks against healthcare workers (261 total). |
| Eloul et al. (29) | Qualitative Case Study | Syria | *2, 3, 4* | - General lack of mental health professionals, practitioners, psychotropic medication. - Need for training for advanced mental health skills, beyond psychological first aid. - General lack of healthcare professionals; forced to flee. |
| Erenel et al. (30) | Quantitative Case-Control Study | Turkey | *1, 2* | - High prevalence of adolescent pregnancies (14%). - Poor antenatal care (41% had no antenatal visits); significantly different from Turkish women. |
| Gammouh et al. (31) | Quantitative Cross-Sectional Study | Jordan | *1* | - Health needs assessment; “pain” (32%), infectious disease (31%), cardiovascular disease (16%). |
| Gammouh et al. (32) | Quantitative Cross-Sectional Study | Jordan | *1* | - High depression (30%) and chronic disease (30%) prevalence |
| Harrison et al. (33) | Qualitative Case Study | Syria | *2, 4* | - Sever destruction of hospitals in the country. - General lack of psychologists; forced to flee. |
| Hornez et al. (34) | Quantitative Case Study | Jordan | *1* | - High prevalence of penetrating injuries (95%) in emergency room. |
| Houssain et al. (35) | Quantitative Cross-Sectional Study | Jordan, Lebanon, and Iraq | *1* | - Malnutrition was below WHO 5% threshold. |
| Huster et al. (36) | Mixed Methods Cross-Sectional Study | Lebanon | *1, 2, 4* | - Deliveries were the most prevalent reason for hospital admission (44%); high number of caesarean sections (35%). - Syrian women reported low antenatal care attendance. - General lack of female health care workers. |
| Ibrahim et al. (37) | Quantitative Case Study | Iraq | *1* | - High prevalence of PTSD (35-38%) |
| Inci et al. (38) | Quantitative Cross-Sectional Study | Turkey | *1* | - Low prevalence of Hep B (3%), Hep C (2%), and HIV (0%) |
| Inci et al. (39) | Quantitative Cross-Sectional Study | Turkey | *1* | - Cutaneous leishmaniosis more prevalent in Syrians (69%) compared to Turkish citizens (31%). |
| Jefee-Bahloul et al. (40) | Quantitative Cross-Sectional Study | Turkey | *1, 2* | - High prevalence of stress cores among Syrians (42%). - High need for mental health service (34% expressed need to see one but currently were not). |
| Karakus et al. (41) | Quantitative Cross-Sectional Study | Turkey | *1* | - From the emergency department; high prevalence of gunshot wounds (70%) and internal injuries (14%). |
| Kazour et al. (42) | Quantitative Cross-Sectional Study | Lebanon | *1* | - High prevalence of PTSD (27% point, 35% lifetime). |
| Kocamer Simsek et al. (43) | Quantitative Cross-Sectional Study | Turkey | *1* | - From the emergency department; high prevalence of gunshot wounds (84%) and shrapnel injuries (16%). |
| Krause et al. (44) | Qualitative Case Study | Jordan | *2* | - Limited access to clinical management for rape victims, quality of obstetrics was criticized by refugees, lack of PHCC in the refugee camps. |
| Lama et al. (45) | Quantitative Case-Control Study | Lebanon | *1* | - High prevalence of schizophrenia (38%), bipolar (17%), depressive disorder (11%), and substance abuse disorders (10%). |
| Makhoul et al. (46) | Quantitative Case Study | Lebanon | *1* | - High rates of preterm births and infants with congenital malformations. |
| Marwa et al. (47) | Quantitative Cross-Sectional Study | Turkey | *1* | - High prevalence of PTSD (61%), anxiety (53%), and depression (27%). |
| Naja et al. (48) | Quantitative Cross-Sectional Study | Lebanon | *1* | - High prevalence of Major Depressive Disorder (44%). |
| Ozkeklikci et al. (49) | Quantitative Case-Control Study | Turkey | *1* | - Cutaneous leishmaniosis is more prevalent in Syrians (66%) compared to Turkish citizens (33%). |
| Parkinson et al. (50) | Qualitative Case Study | Lebanon | *2* | - Refugees could not access care to informational barriers, were subject to prejudice at the hospitals. |
| Reese Masterson et al. (51) | Quantitative Cross-Sectional Study | Lebanon | *1, 2* | - Poor reproductive health; 54% menstrual irregularities, 53% RTI infection; delivery complications (40%). - Reproductive health viewed as unavailable (45%) or inaccessible (40%); main barrier was price (50%). |
| Roberton et al. (52) | Quantitative Cross-Sectional Study | Jordan and Lebanon | *1, 2* | - Jordan: 39% vaccination rates; difficulty obtaining vaccinations (34%) - Lebanon: 20% vaccination rates; difficulty obtaining vaccinations (40%). |
| Rossi et al. (53) | Quantitative Case-Control Study | Lebanon | *1, 2* | - High vaccination rates but not enough; polio (84%), pentavalent (72%), MMR (61%). - Low rate of fully vaccinated children (54%). |
| Salhool et al. (54) | Mixed Methods Case Study | Turkey | *2, 3* | - Number of refugee cases seen each day exceeds the recommended limit; insufficient rehabilitation and child psychology services. - Need for skills in communication (Arabic). |
| Saroufim et al. (55) | Quantitative Cross-Sectional Study | Lebanon | *1* | - High prevalence of cutaneous leishmaniosis among Syrian refugees. |
| Savas et al. (56) | Quantitative Cross-Sectional Study | Turkey | *1, 2, 3, 4* | - Health needs assessment by hospital admissions: Gunshot wounds most common (62%); NCDs, nosocomial infection (46%), viral hepatitis (42%) and TB (36%). - Difficulty communicating with refugee patients. - Need for skills in communication (Arabic). - General lack of healthcare professionals; resignation, overworked. |
| Sekkarie et al. (57) | Qualitative Case Study | Syria | *2, 3, 4* | - 52% of hemodialysis centers are operational. - Need for skills to properly operate a dialysis machine. - Lack of nephrologists; forced to flee. |
| Sevinc et al. (58) | Qualitative Case Study | Turkey | *2, 3* | - Difficulty communicating with payments (language gap). - Need for skills to properly operate a dialysis machine. |
| Simsek et al. (59) | Quantitative Cross-Sectional Study | Turkey | *1* | - Women’s needs assessment: high prevalence of mental health symptoms (90% reported 2 symptoms), iron and B12 deficiencies (50% and 46%), and STI symptoms (51%). |
| Strong et al. (60) | Mixed Methods Cross-Sectional Study | Lebanon | *1, 2* | - Health needs assessment of older populations: hypertension (60%), diabetes (47%), heart disease (30%). - Financial barriers cited as the main reason for not seeking health care (79%). |
| Tahirbegolli et al. (61) | Quantitative Cross-Sectional Study | Turkey | *1* | - Hospital health needs assessment: Respiratory diseases (14%), and eye diseases (13%); most common use is the emergency department (29%), pediatric (26%), ophthalmology (10%). |
| Tappis et al. (62) | Quantitative Cross-Sectional Study | Jordan and Lebanon | *1, 2* | - Jordan: 21% of households reported a birth, averaging on 6.2 antenatal visits. - Lebanon: 22% of households reported a birth, averaging on 4.8 antenatal visits. - Cost cited as the primary barrier for not seeking delivery location. |
| West et al. (63) | Qualitative Case Study | Jordan | *2* | - Poor knowledge of available services and low prioritization of family planning; need for more female staff. |

1. Abo-Hilal M, Hoogstad M. Syrian mental health professionals as refugees in Jordan: establishing mental health services for fellow refugees. Intervention (15718883). 2013;11(1):89-93.

2. Acarturk C, Cetinkaya M, Senay I, Gulen B, Aker T, Hinton D. Prevalence and Predictors of Posttraumatic Stress and Depression Symptoms Among Syrian Refugees in a Refugee Camp. Journal of Nervous and Mental Disease. 2017.

3. Akoury-Dirani L, Sahakian TS, Hassan FY, Hajjar RV, El Asmar K. Psychological first aid training for Lebanese field workers in the emergency context of the Syrian refugees in Lebanon. Psychological trauma : theory, research, practice and policy. 2015;7(6):533-8.

4. Al Qadire M, Aljezawi M, en, Al-Shdayfat N. Cancer Awareness and Barriers to Seeking Medical Help Among Syrian Refugees in Jordan: a Baseline Study. 2017.

5. Alawieh A, Musharrafieh U, Jaber A, Berry A, Ghosn N, Bizri AR. Revisiting leishmaniasis in the time of war: The Syrian conflict and the Lebanese outbreak. International Journal of Infectious Diseases. 2014;29:115-9.

6. Al-Fahoum AS, Diomidous M, Mechili A, Archangelidi O, Theodoromanolakis P, Mantas J. The Provision of Health Services in Jordan to Syrian Refugees. Health Science Journal. 2015;9(2):1-7.

7. Alnuaimi K, Kassab M, Ali R, Mohammad K, Shattnawi K. Pregnancy outcomes among Syrian refugee and Jordanian women: a comparative study. 2017.

8. Alpak G, Unal A, Bulbul F, Sagaltici E, Bez Y, Altindag A, et al. Post-traumatic stress disorder among Syrian refugees in Turkey: A cross-sectional study. International Journal of Psychiatry in Clinical Practice. 2015;19(1):45-50.

9. Al-Smadi AM, Halaseh HJ, Gammoh OS, Ashour AF, Gharaibeh B, Khoury LS. Do chronic diseases and availability of medications predict post-traumatic stress disorder (PTSD) among Syrian refugees in Jordan? Pakistan Journal of Nutrition. 2016;15(10):936-41.

10. Ay M, Arcos Gonzalez P, Castro Delgado R. The Perceived Barriers of Access to Health Care Among a Group of Non-camp Syrian Refugees in Jordan. International Journal of Health Services. 2016;46(3):566-89.

11. Basheti IA, Qunaibi EA, Malas R. Psychological impact of life as refugees: A pilot study on a Syrian Camp in Jordan. Tropical Journal of Pharmaceutical Research. 2015;14(9):1695-701.

12. Benage M, Greenough PG, Vinck P, Omeira N, Pham P. An assessment of antenatal care among Syrian refugees in Lebanon. Confl Health. 2015;9:8-.

13. Bilukha OO, Jayasekaran D, Burton A, Faender G, King, ori J, et al. Nutritional status of women and child refugees from Syria-Jordan, April-May 2014. MMWR Morb Mortal Wkly Rep. 2014;63(29):638-9.

14. Bouchghoul H, Hornez E, Duval-Arnould X, Philippe HJ, Nizard J. Humanitarian obstetric care for refugees of the Syrian war. the first 6 months of experience of Gynécologie Sans Frontières in Zaatari Refugee Camp (Jordan). Acta Obstetricia et Gynecologica Scandinavica. 2015;94(7):755-9.

15. Bucak IH, Almis H, Benli S, Turgut M. An overview of the health status of Syrian refugee children in a tertiary hospital in Turkey. Avicenna journal of medicine. 2017;7(3):110-4.

16. Cartwright K, El-Khani A, Subryan A, Calam R. Establishing the feasibility of assessing the mental health of children displaced by the Syrian conflict. Global Mental Health. 2015;2:e8.

17. Cetorelli V, Burnham G, Shabila N. Prevalence of non-communicable diseases and access to health care and medications among Yazidis and other minority groups displaced by ISIS into the Kurdistan Region of Iraq. Confl Health. 2017;11:4-.

18. Chemali Z, Borba CPC, Johnson K, Hock RS, Parnarouskis L, Henderson DC, et al. Humanitarian space and well-being: effectiveness of training on a psychosocial intervention for host community-refugee interaction. Medicine, conflict, and survival. 2017:1-21.

19. Cherri Z, Cuesta JG, Rodriguez-Llanes JM, Guha-Sapir D. Early marriage and barriers to contraception among syrian refugee women in lebanon: A qualitative study. International Journal of Environmental Research and Public Health. 2017;14(8).

20. Chung MC, AlQarni N, Al Muhairi S, Mitchell B. The relationship between trauma centrality, self-efficacy, posttraumatic stress and psychiatric co-morbidity among Syrian refugees: Is gender a moderator? Journal of Psychiatric Research. 2017;94:107-15.

21. Collins DRJ, Jobanputra K, Frost T, Muhammed S, Ward A, Shafei AA, et al. Cardiovascular disease risk and prevention amongst Syrian refugees: mixed methods study of Medecins Sans Frontieres programme in Jordan. Conflict & Health [Electronic Resource]. 2017;11:14.

22. Demirci H, Yildirim Topak N, Ocakoglu G, Karakulak Gomleksiz M, Ustunyurt E, Ulku Turker A. Birth characteristics of Syrian refugees and Turkish citizens in Turkey in 2015. International Journal of Gynecology and Obstetrics. 2017;137(1):63-6.

23. Doocy S, Lyles E, Akhu-Zaheya L, Burton A, Burnham G. Health service access and utilization among Syrian refugees in Jordan. International Journal for Equity in Health. 2016;16:1-15.

24. Doocy S, Lyles E, Hanquart B, Team LS, Woodman M. Prevalence, care-seeking, and health service utilization for non-communicable diseases among Syrian refugees and host communities in Lebanon. Conflict & Health [Electronic Resource]. 2016;10:21.

25. Doocy S, Lyles E, Roberton T, Akhu-Zaheya L, Oweis A, Burnham G. Prevalence and care-seeking for chronic diseases among Syrian refugees in Jordan. BMC Public Health. 2015;15(1):1-10.

26. Doocy S, Paik K, Lyles E, Tam HH, Fahed Z, Winkler E, et al. Pilot Testing and Implementation of a mHealth tool for Non-communicable Diseases in a Humanitarian Setting. PLoS currents. 2017;9:05.

27. Duzkoylu Y, Basceken SI, Kesilmez EC. Physical Trauma among Refugees: Comparison between Refugees and Local Population Who Were Admitted to Emergency Department-Experience of a State Hospital in Syrian Border District. Journal of Environmental & Public Health. 2017:1-5.

28. Elamein M, Bower H, Valderrama C, Zedan D, Rihawi H, Almilaji K, et al. Attacks against health care in Syria, 2015-16: results from a real-time reporting tool. Lancet. 2017.

29. Eloul L, Quosh C, Ajlami R, Avetisyan N, Barakat M, Barakat L, et al. Inter-agency coordination of mental health and psychosocial support for refugees and people displaced in Syria. Intervention (15718883). 2013;11(3):340-8.

30. Erenel H, Aydogan Mathyk B, Sal V, Ayhan I, Karatas S, Koc Bebek A. Clinical characteristics and pregnancy outcomes of Syrian refugees: a case–control study in a tertiary care hospital in Istanbul, Turkey. Archives of Gynecology and Obstetrics. 2017;295(1):45-50.

31. Gammoh OS. A preliminary description of medical complaints and medication consumption among 375 Syrian refugees residing in North Jordan. Jordan Journal of Pharmaceutical Sciences. 2016;9(1):13-21.

32. Gammouh OS, Al-Smadi AM, Tawalbeh LI, Khoury LS. Chronic diseases, lack of medications, and depression among Syrian refugees in Jordan, 2013-2014. Preventing Chronic Disease. 2015;12:E10-E.

33. Harrison S, Dahman R, Ismail M, Saada E, Hassan M, Hassan R, et al. 'Against all odds': UNHCR's mental health and psychosocial support programme for Iraqi refugees and internally displaced Syrians. Intervention (15718883). 2013;11(2):190-4.

34. Hornez E, Ramiara P, Mocellin N, Bajard X, Legoudeveze S, Charpail C, et al. Surgical management of Syria's war casualties: experience from a French surgical team deployed in the Zaatari refugee camp (Jordan). European journal of trauma and emergency surgery : official publication of the European Trauma Society. 2015;41(2):143-7.

35. Hossain SM, Leidman E, Kingori J, Al Harun A, Bilukha OO. Nutritional situation among Syrian refugees hosted in Iraq, Jordan, and Lebanon: cross sectional surveys. Conflict & Health [Electronic Resource]. 2016;10:26.

36. Huster KM, Patterson N, Schilperoord M, Spiegel P. Cesarean sections among Syrian refugees in Lebanon from december 2012/january 2013 to june 2013: probable causes and recommendations. Yale Journal of Biology & Medicine. 2014;87(3):269-88.

37. Ibrahim H, Hassan CQ. Post-traumatic Stress Disorder Symptoms Resulting from Torture and Other Traumatic Events among Syrian Kurdish Refugees in Kurdistan Region, Iraq. Front Psychol. 2017;8:241-.

38. Inci A, Sarici IS, Çalişkan G, Kalayci MU. Investigation of frequency of HBSAG, anti HBS, anti HCV and anti HIV in refugee patients from Syria who admit to a training and research hospital department of surgery. Acta Medica Mediterranea. 2017;33(1):59-63.

39. Inci R, Ozturk P, Mulayim MK, Ozyurt K, Alatas ET, Inci MF. Effect of the Syrian civil war on prevalence of cutaneous leishmaniasis in Southeastern Anatolia, Turkey. Medical Science Monitor. 2015;21:2100-4.

40. Jefee-Bahloul H, Moustafa MK, Shebl FM, Barkil-Oteo A. Pilot assessment and survey of Syrian refugees' psychological stress and openness to referral for telepsychiatry (PASSPORT Study). Telemedicine journal and e-health : the official journal of the American Telemedicine Association. 2014;20(10):977-9.

41. Karakus A, Yengil E, Akkucuk S, Cevik C, Zeren C, Uruc V. The reflection of the Syrian civil war on the emergency department and assessment of hospital costs. Ulusal Travma ve Acil Cerrahi Dergisi = Turkish Journal of Trauma & Emergency Surgery: TJTES. 2013;19(5):429-33.

42. Kazour F, Zahreddine NR, Maragel MG, Almustafa MA, Soufia M, Haddad R, et al. Post-traumatic stress disorder in a sample of Syrian refugees in Lebanon. Comprehensive Psychiatry. 2017;72:41-7.

43. Kocamer Şimşek B, Dokur M, Uysal E, Çalıker N, Gökçe ON, Deniz İ K, et al. Characteristics of the injuries of syrian refugees sustained during the civil war. Ulusal Travma ve Acil Cerrahi Dergisi. 2017;23(3):199-206.

44. Krause S, Williams H, Onyango MA, Sami S, Doedens W, Giga N, et al. Reproductive health services for Syrian refugees in Zaatri Camp and Irbid City, Hashemite Kingdom of Jordan: an evaluation of the Minimum Initial Services Package. Confl Health. 2015;9(Suppl 1 Taking Stock of Reproductive Health in Humanitarian):S4-S.

45. Lama S, François K, Marwan Z, Sami R. Impact of the Syrian Crisis on the Hospitalization of Syrians in a Psychiatric Setting. Community mental health journal. 2016;52(1):84-93.

46. Makhoul G, Falakha G, Makhoul CN, Ahad AA. Impact of Syrian refugees on neonatal care in Hopital Notre dame de la paix, Akkar, north Lebanon. NeoReviews. 2015;16(8):e456-e61.

47. Marwa KI. Psychosocial sequels of syrian conflict. African Journal of Psychiatry (South Africa). 2016;19(2).

48. Naja WJ, Aoun MP, El Khoury EL, Abdallah FJ, Haddad RS. Prevalence of depression in Syrian refugees and the influence of religiosity. Comprehensive Psychiatry. 2016;68:78-85.

49. Ozkeklikci A, Karakus M, Ozbel Y, Toz S. The new situation of cutaneous leishmaniasis after Syrian civil war in Gaziantep city, Southeastern region of Turkey. Acta Tropica. 2017;166:35-8.

50. Parkinson SE, Behrouzan O. Negotiating health and life: Syrian refugees and the politics of access in Lebanon. Social Science & Medicine. 2015;146:324-31.

51. Reese Masterson A, Usta J, Gupta J, Ettinger AS. Assessment of reproductive health and violence against women among displaced Syrians in Lebanon. BMC Women's Health. 2014;14(1):25-.

52. Roberton T, Weiss W, Jordan Health Access Study T, Lebanon Health Access Study T, Doocy S. Challenges in Estimating Vaccine Coverage in Refugee and Displaced Populations: Results From Household Surveys in Jordan and Lebanon. Vaccines. 2017;5(3).

53. Rossi R, Assaad R, Rebeschini A, Hamadeh R. Vaccination coverage cluster surveys in middle Dreib - Akkar, Lebanon: Comparison of vaccination coverage in children aged 12-59 months Pre-and post-vaccination campaign. PLoS ONE. 2016;11(12).

54. Sahlool Z, Sankri-Tarbichi AG, Kherallah M. Evaluation report of health care services at the Syrian refugee camps in Turkey. Avicenna J Med. 2012;2(2):25-8.

55. Saroufim M, Charafeddine K, Issa G, Khalifeh H, Habib RH, Berry A, et al. Ongoing epidemic of cutaneous leishmaniasis among Syrian refugees, Lebanon. Emerging Infectious Diseases. 2014;20(10):1712-5.

56. Savas N, Arslan E, İnandı T, Yeniçeri A, Erdem M, Kabacaoğlu M, et al. Syrian refugees in Hatay/Turkey and their influence on health care at the university hospital. International Journal of Clinical and Experimental Medicine. 2016;9(9):18281-90.

57. Sekkarie MA, Zanabli AR, Rifai AO, Murad LB, Al-Makki AA. The Syrian conflict: Assessment of the ESRD system and response to hemodialysis needs during a humanitarian and medical crisis. Kidney International. 2015;87(2):262-5.

58. Sevinc S, Kilic SP, Ajghif M, Ozturk MH, Karadag E. Difficulties encountered by hospitalized Syrian refugees and their expectations from nurses. International Nursing Review. 2016;63(3):406-14.

59. Simsek Z, Yentur Doni N, Gul Hilali N, Yildirimkaya G. A community-based survey on Syrian refugee women's health and its predictors in Sanliurfa, Turkey. Women & Health. 2017:1-15.

60. Strong J, Varady C, Chahda N, Doocy S, Burnham G. Health status and health needs of older refugees from Syria in Lebanon. Confl Health. 2015;9:12-.

61. Tahirbegolli B, Cavdar S, Cetinkaya Sumer E, Akdeniz SI, Vehid S. Outpatient admissions and hospital costs of Syrian refugees in a Turkish university hospital. Saudi Medical Journal. 2016;37(7):809-12.

62. Tappis H, Lyles E, Burton A, Doocy S. Maternal Health Care Utilization Among Syrian Refugees in Lebanon and Jordan. 2017.

63. West L, Isotta-Day H, Ba-Break M, Morgan R. Factors in use of family planning services by Syrian women in a refugee camp in Jordan. 2016.
