# Supplementary material for: Assessment of the health needs of Syrian refugees in Lebanon and Syria’s neighboring countries
Source: Confl Health. 2019 Jun 27;13:31. doi: 10.1186/s13031-019-0211-3 (PMC6598365; doi:10.1186/s13031-019-0211-3)
Supplement: Supplementary file 6 — Table S3. MMAT Risk of Bias Summary. (DOCX 110 kb) [file 13031_2019_211_MOESM6_ESM.docx]

**Table S3. MMAT Risk of Bias Summary**

| Study ID | Main Limitations | MMAT Rating (out of ****) |
| --- | --- | --- |
| Abo-Hilal et al. [1] | - Did not consider how findings relate to the context in which the data was collected. - Did not consider how findings relate to the researchers influence. | ****** |
| Acarturk et al. [2] | - Sample was not representative. | ******* |
| Akoury-Dirani et al. [3] | - Unacceptable response rate. | ******* |
| Al Qadire et al. [4] | - Sampling strategy was not relevant to research question. | ******** |
| Alawieh et al. [5] |  | ******** |
| Al-Fahoum et al. [6] | - Sampling strategy was not relevant to the research question | ******* |
| Alnuaimi et al. [7] | - Sample was not representative of the research question. | ******* |
| Alpak et al. [8] |  | ******** |
| Al-Smadi et al. [9] |  | ******** |
| Ammar et al. [10] | - Did not consider how findings relate to the context in which the data was collected. | ******* |
| Ay et al. [11] | - Sample not representative of the population. - Did not report the response rate. | ****** |
| Basheti et al. [12] | - Sampling strategy was not relevant to the research question. - Sample is not representative of the population under study. - Did not report the response rate. | ***** |
| Benage et al. [13] | - Sample not representative of the population. | ******* |
| Bilukha et al. [14] | - Sample not representative of the population. - Did not report the response rate. | ****** |
| Bouchghoul et al. [15] | - Sample not representative of the population. | ******* |
| Bucak et al. [16] | - Sample not representative of the population. | ******* |
| Cartwright et al. [17] | - Sample not representative of the population. - Did not report the response rate. | ****** |
| Cetorelli et al. [18] |  | ******** |
| Chemali et al. [19] |  | ******** |
| Cherri et al. [20] | - Did not consider how findings relate to the context in which the data was collected. | ******* |
| Chung et al. [21] | - Sample not representative of the population. - Did not report the response rate. | ****** |
| Collins et al. [22] | - Did not consider how findings relate to the researchers influence. | ******* |
| Demirci et al. [23] |  | ******** |
| Doocy et al. [24] |  | ******** |
| Doocy et al. [25] |  | ******** |
| Doocy et al. [26] |  | ******** |
| Doocy et al. [27] |  | ******** |
| Duzkoylu et al. [28] |  | ******** |
| Elamein et al. [29] | - Sample not representative of the population. | ******* |
| Eloul et al. [30] | - Process for analyzing qualitative data irrelevant to the research question. - Did not consider how findings relate to the context in which the data was collected. - Did not consider how findings relate to the researchers influence. - Note: Mapping study – difficult to assess quality using the MMAT. | ****** |
| Erenel et al. [31] |  | ******** |
| Gammouh et al. [32] |  | ******** |
| Gammouh et al. [33] |  | ******** |
| Harrison et al. [34] | - Process for analyzing qualitative data irrelevant to the research question. - Did not consider how findings relate to the context in which the data was collected. - Did not consider how findings relate to the researchers influence. | ***** |
| Hornez et al. [35] |  | ******** |
| Houssain et al. [36] |  | ******** |
| Huster et al. [37] | - Did not appropriately consider the limitations associated with integration of qualitative and quantitative data. | ******* |
| Ibrahim et al. [38] |  | ******** |
| Inci et al. [39] |  | ******** |
| Inci et al. [40] | - Sampling strategy not relevant to address the research question. - Sample not representative of the population. | ****** |
| Jefee-Bahloul et al. [41] | - Sample not representative of the research question. - Response rate not reported. | ****** |
| Karakus et al. [42] | - Sampling strategy not relevant to address the research question. - Sample not representative of the population. | ***** |
| Kazour et al. [43] |  | ******** |
| Kocamer Simsek et al. [44] | - Sample not representative of the population. | ****** |
| Krause et al. [45] |  | ******** |
| Lama et al. [46] |  | ******** |
| Makhoul et al. [47] | - Sample not representative of the population. - Measurements inappropriate. | ***** |
| Marwa et al. [48] | - Sample not representative of the population. - Not an acceptable response rate. | ****** |
| Naja et al. [49] |  | ******** |
| Ozkeklikci et al. [50] | - Sample not representative of the population. | ******* |
| Parkinson et al. [51] |  | ******** |
| Reese Masterson et al. [52] |  | ******** |
| Roberton et al. [53] |  | ******** |
| Rossi et al. [54] |  | ******** |
| Salhool et al. [55] | - Process for analyzing qualitative data not relevant to the research question. - Did not consider how findings relate to the context in which the data was collected. - Did not consider how findings relate to the researchers influence. - Note: Report, difficult to assess quality. | ****** |
| Saroufim et al. [56] | - Sampling strategy not relevant to the research question. - Sample not representative of the population. | ***** |
| Savas et al. [57] | - Sample not representative of the population. - Did not report response rate. | ****** |
| Sekkarie et al. [58] | - Did not consider how findings relate to the context in which the data was collected. - Did not consider how findings relate to the researchers influence. | ****** |
| Sevinc et al. [59] |  | ******** |
| Simsek et al. [60] |  | ******** |
| Strong et al. [61] | - Process for analyzing qualitative data not relevant to the research question. - Did not consider how findings relate to the researchers influence. | ****** |
| Tahirbegolli et al. [62] |  | ******** |
| Tappis et al. [63] |  | ******** |
| West et al. [64] | - Did not consider how findings relate to the researchers influence. | ******* |
