# Supplementary figures and images for: Assessment of the health needs of Syrian refugees in Lebanon and Syria’s neighboring countries
Source: Confl Health. 2019 Jun 27;13:31. doi: 10.1186/s13031-019-0211-3 (PMC6598365; doi:10.1186/s13031-019-0211-3)

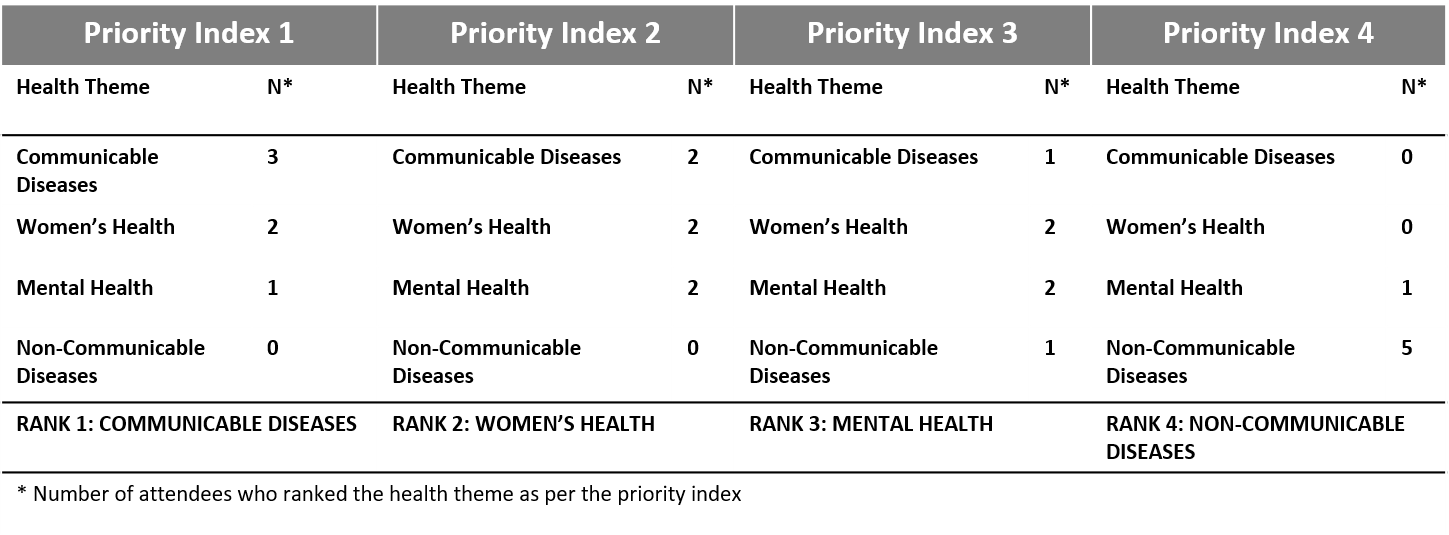

Supplement: Supplementary file 7 — Results of the validation meeting on health needs prioritization, conducted with the directors and representatives of MOPH PHCs. (PNG 39 kb) [file 13031_2019_211_MOESM7_ESM.png]
